# Supplementary material for: Darwin Assembly: fast, efficient, multi-site bespoke mutagenesis
Source: Nucleic Acids Res. 2018 Feb 2;46(8):e51. doi: 10.1093/nar/gky067 (PMC5934624; doi:10.1093/nar/gky067)
Supplement: Supplementary Data [file gky067_supp.zip › nar-03248-met-g-2017-File008.docx]

**Darwin Assembly: fast, efficient, multi-site bespoke mutagenesis.**

Christopher Cozens^1^ and Vitor B. Pinheiro*^1,2^

^1^ University College London, Gower Street, London WC1E 6BT, UK

^2^ Institute of Structural and Molecular Biology, Birkbeck College, University of London, Malet Street, WC1E 7HX, UK

* To whom correspondence should be addressed. Tel: +44 (0)20 7679 4481; Email: v.pinheiro@ucl.ac.uk

**SUPPLEMENTARY INFORMATION**

| Figure S1 | **Darwin Assembly boundary oligonucleotide architecture.** |
| --- | --- |
| Figure S2 | **Assembly of KOD 6G12** |
| Figure S3 | **Design of CAT alanine scanning and indel libraries.** |
| Table S1 | **Oligonucleotides used in this study.** |
| Table S2 | **Observed codon frequencies for single NNS insertion between CAT Phe102 and Ser103 (22611 reads).** |
| Table S3 | **Observed codon frequencies for double NNS insertion between CAT Phe102 and Ser103 (22611 reads).** |
| Table S4 | **Relative CAT deletion frequencies.** |
| Table S5 | **Predicted effect of deletions on primer mutagenic primer annealing** |
| Table S6 | **T7RSS library generation Miseq data.** |
| Table S7 | **TgoT library generation Miseq data for 20 codon diversity.** |
| Table S8 | **TgoT library generation Miseq data for reduced diversity mutagenesis.** |
| Supplementary Protocol 1 | **T7RSS library using biotinylated oligonucleotide and streptavidin cleanup, and critical experimental considerations** |
| Supplementary Protocol 2 | **TgoT library using theta oligonucleotide and exonuclease cleanup** |
| Supplementary Protocol 3 | **Codon frequency counting** |


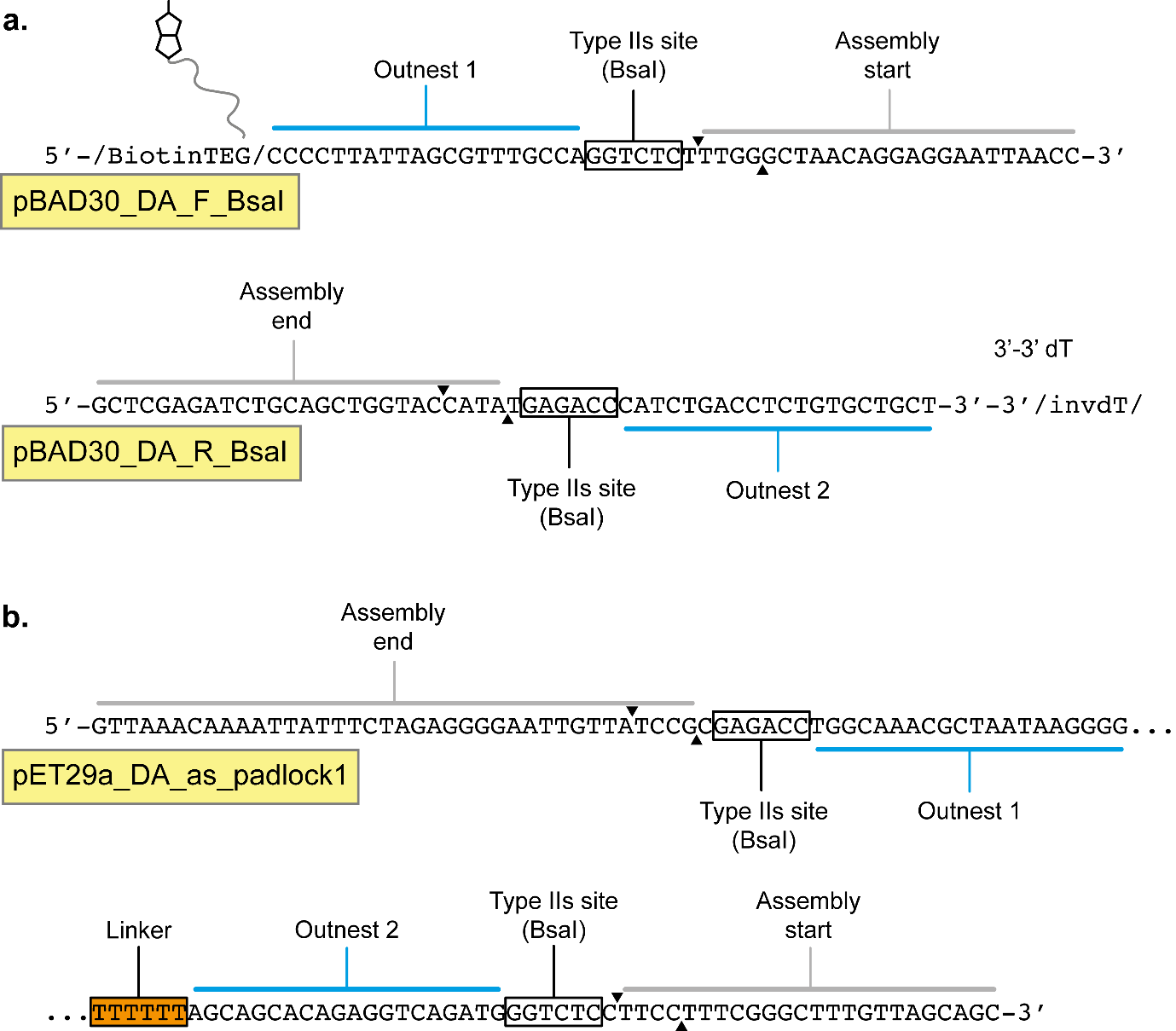


**Figure S1: Darwin Assembly boundary oligonucleotide architecture.** (a) Design of 2-primer assembly (as described in Figure 1). pBAD30_DA_F_BsaI is the 5’-boundary oligonucleotide used for T7RSS mutagenesis. It is 5’-biotinylated and encodes priming sites (annotated above the sequence) for outnest PCR (blue) and assembly start (grey). The Type IIs recognition site is place between the two priming sites to enable Golden Gate assembly. pBAD30_DA_R_BsaI is the 3’-boundary oligonucleotide used for T7RSS mutagenesis. It encodes the binding site for the assembly end and the reverse complement of Type IIs recognition site and outnest primer used in the downstream amplification. Annotation of reverse complement sequences are shown below the sequence for clarity. This architecture uses the 3’-boundary oligonucleotide to terminate the assembly. Primers shown were used in the assembly of T7RSS libraries. (b) Design of theta assembly oligonucleotide (described in Figure 2). A single oligoucleotide (pET29a_DA_as_padlcok1) was used in the assembly of the TgoT library. Both 5’ and 3’ regions of this oligonucleotide anneal to the plasmid during assembly: the 3’ region as the primer and the 5’ region as the termination point. Between these regions BsaI sites and outnesting priming sites are encoded, linked by a flexible poly(dT) linker (orange), The strand assembled for the TgoT library was the antisense strand and the orientation of the outnest primers was swapped. Oligonucleotide names are shown in yellow boxes and BsaI cut sites are shown as black triangles. Arrows above the sequence represent cuts of the strand being presented, and arrows below the sequence represent the position of the cut sites in the reverse complementary strand generated after the outnest PCR.


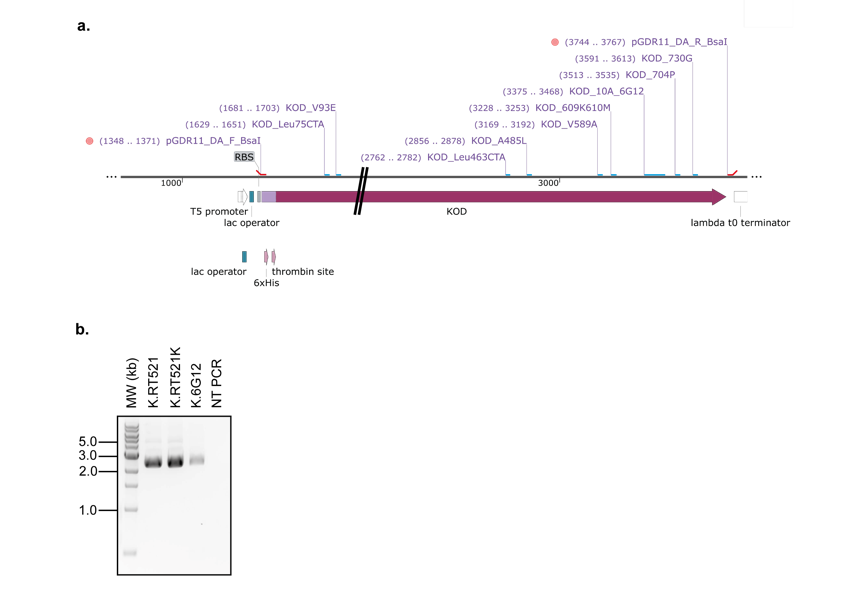


**Figure S2: Darwin assembly of KOD 6G12.** (a) Mutations previously reported in *Thermococcus gorgonarius* DNA polymerase to enable 1,5-anhydrohexitol nucleic acid synthesis (22) were mapped to the homologous *Thermococcus kodakarensis* DNA polymerase and introduced by Darwin assembly. Assembly used 2 boundary oligonucleotides (red) and 9 mutagenic oligonucleotides (blue) encoding the necessary 38 point mutations (shown in SI Table 1). (b) Post assembly amplification of KOD 6G12 (K.6G12) and other enzyme variants implicated in xenobiotic nucleic acid synthesis and reverse transcription (22). Expected assembly length 2378 bp and a no template control was included in the experiment (NT PCR). Transformants were isolated and sequence-verified to confirm incorporation of all targeted mutations.


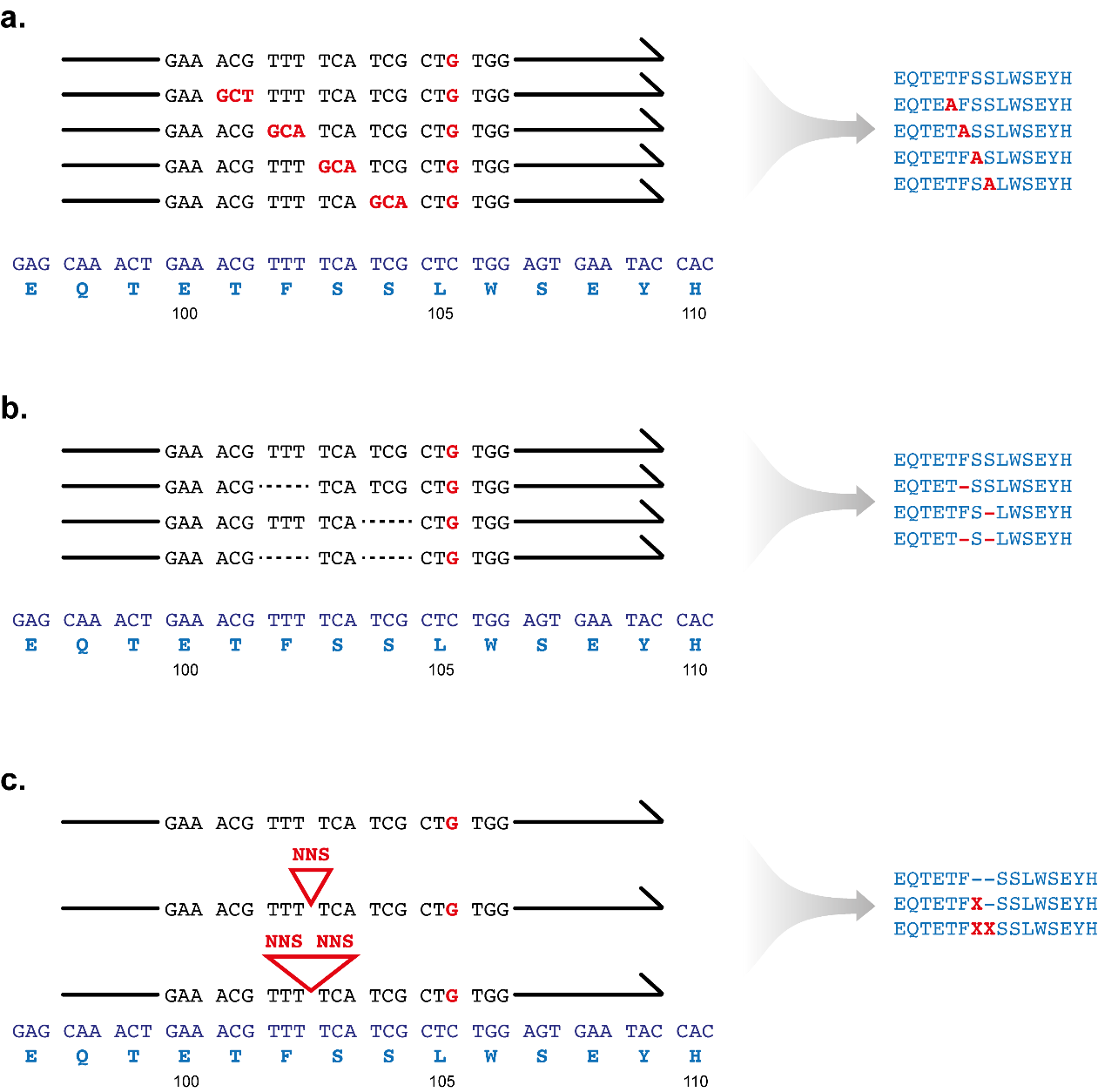


**Figure S3: Design of CAT alanine scanning and indel libraries.** All oligonucleotides were designed to bind to the same region of CAT gene, with no attempt to adjust overall melting temperature by extending flanking sequences. (a) Five inner oligonucleotides were designed for the synthesis of the alanine scan library, all introducing the control Leu105(CTC🠚CTG) mutation (and four of them introducing an alanine at one of Thr101, Phe102, Ser103 or Ser104. The oligonucleotides were mixed in equimolar ratio, so all variants were expected at 20%. (b) For the deletion library, four oligonucleotides were designed. All introduced the control Leu105(CTC🠚CTG) mutation and three of them introduced either single (Phe102 or Ser104) or double (Phe102 and Ser104) mutations. It is likely that the double deletion oligo, resulted in deletion of Phe102 and Ser103 with Ser104 being recoded. (c) For the insertion library, three oligonucleotides were designed introducing Leu105(CTC🠚CTG) and up to two NNS incorporations between Phe102 and Ser103.

**Table S1: Oligonucleotides used in this study.** See Figure S1 for explanation of outer oligonucleotide architecture. Note that TgoT mutations are on the antisense strand. TgoT_490RSC series actually encode SRC (the correct antisense codon would be GSY).

| **Name** | **Sequence (5’-3’)** | **Function** |
| --- | --- | --- |
| PCR primers |  |  |
| Outnest 1 | CCCCTTATTAGCGTTTGCCA | Primer pair used to for outnest PCR of nascent strand after assembly. |
| Outnest 2 | AGCAGCACAGAGGTCAGATG |  |
| pSB1C3_Amp_vec_F | GGTGCCTCACTGATTAAGCATTGGTAAcagaaatcatccttagcgaaagctaagg | Primer pair to make pSB1C3 vector for ampicillin cloning. |
| pSB1C3_Amp_vec_R | GAATGTATTTAGAAAAATAAACAAATAGGGGTTCCGCGttacttcgcgttatgcaggcttc |  |
| pSB1C3_Amp_frag_F | gaagcctgcataacgcgaagtaaCGCGGAACCCCTATTTGTTTATTTTTCTAAATACATTC | Primer pair to amplify beta lactamase from pUC19 for construction of pSB1C3A. |
| pSB1C3_Amp_frag_R | ccttagctttcgctaaggatgatttctgTTACCAATGCTTAATCAGTGAGGCACC |  |
| CAT_136R_BsaI | gagtcaGGTCTCaggccgtaatatccagctgaacgg | Primer pair to make CAT vector from pSB1C3A2. |
| CAT_651F_BsaI | gagtcaGGTCTCcgatgagtggcagggcg |  |
| pET_assembly_vec_F | gagtcaggtctcAGGAAGCTGAGTTGGCTGCTG | Primer pair to make vector for TgoT assembly from pET29aΔΔΔ. |
| pET_assembly_vec_R | gagtcaggtctcATCCGCTCACAATTCCCCTATAGTGAG |  |
| pBAD30_DA_vec_F2BsaI | GAGTCAGGTCTCCCATATGGGAATTCGAAGCTTGG | Primer pair to make vector for T7RSS assembly from pBAD30. |
| pBAD30_DA_vec_R2BsaI | GAGTCAGGTCTCCCCAAAAAACGGGTATGGAG |  |
| pGDR11_DA_vec_R_BsaI | GAGTCAGGTCTCCATAGTTAATTTCTCCTCTTTAATGAATTC | Primer pair to make vector for KOD assembly from pGDR11. |
| pGDR11_DA_vec_F_BsaI | GAGTCAGGTCTCTAGCTGAGCTTGGAC |  |
|  |  |  |
| Inner oligonucleotides (CAT) | | |
| CAT_L63CTG | ctttattcacattCTGgcccgcctgatg | Introduce mutations to CAT. |
| CAT_L105CTG | cgttttcatcgCTGtggagtgaatac |  |
| CAT_L117CTG | tccggcagtttCTGcacatatattc |  |
| CAT_L158CTG | ccagttttgatCTGaacgtggccaatatg |  |
| CAT_L105CTG2 | caaactgaaacgttttcatcgctGtggagtgaatac | CAT alanine scanning experiment. One of Thr101, Phe103, Ser103 and Ser104 is mutated to Ala. Silent Leu105->CTG is always included as a positive control. |
| CAT_T101A,L105CTG | caaactgaaGCTttttcatcgctGtggagtgaatac |  |
| CAT_F102A,L105CTG | caaactgaaacgGCAtcatcgctGtggagtgaatac |  |
| CAT_S103A,L105CTG | caaactgaaacgtttGCAtcgctGtggagtgaatac |  |
| CAT_S104A,L105CTG | caaactgaaacgttttcaGCActGtggagtgaatac |  |
| CAT_F102del,L105CTG | caaactgaaacgtcatcgctGtggagtgaatac | CAT deletion experiment. Phe102, Ser104 or both Phe102 and Ser104 are deleted. Silent Leu105->CTG is always included as a positive control. |
| CAT_S104del,L105CTG | caaactgaaacgttttcactGtggagtgaatac |  |
| CAT_F102del,S104del,L105CTG | caaactgaaacgtcactGtggagtgaatac |  |
| CAT_103NNS,L105CTG | caaactgaaacgttttNNScatcgctGtggagtgaatac | CAT insertion experiment. 1 random codon (NNS) or 2 random codons (NNSNNS) are inserted between Phe102 and Ser103. Silent Leu105->CTG is always included as a positive control. |
| CAT_103NNSNNS,L105CTG | caaactgaaacgttttNNSNNScatcgctGtggagtgaatac |  |
|  |  |  |
| Inner oligonucleotides (Tgo) | | |
| TgoT_384YWC,389NDT | ggccacgttccggttctttAHNgtaaccaccggcGWRactctcgcgacg | Tgo library generation. These oligonucleotides are mixed at specific ratios to introduce defined mutations to Tgo. Note they anneal to the sense strand and hence are the reverse complement sequence. |
| TgoT_384YWC,389VMA | ggccacgttccggttctttTKBgtaaccaccggcGWRactctcgcgacg |  |
| TgoT_384YWC,389ATG | ggccacgttccggttctttCATgtaaccaccggcGWRactctcgcgacg |  |
| TgoT_384YWC,389TGG | ggccacgttccggttctttCCAgtaaccaccggcGWRactctcgcgacg |  |
| TgoT_490RSC,493NDT | gtaataaccataAHNactattGYScagaatcttg |  |
| TgoT_490RSC,493VMA | gtaataaccataTKBactattGYScagaatcttg |  |
| TgoT_490RSC,493ATG | gtaataaccataCATactattGYScagaatcttg |  |
| TgoT_490RSC,493TGG | gtaataaccataCCAactattGYScagaatcttg |  |
| TgoT_F587NDT | cttggtaacgaaAHNcccgcgtttg |  |
| TgoT_F587VMA | cttggtaacgaaTKBcccgcgtttg |  |
| TgoT_F587ATG | cttggtaacgaaCATcccgcgtttg |  |
| TgoT_F587TGG | cttggtaacgaaCCAcccgcgtttg |  |
| TgoT_E664NDT | gggtgatctgAHNatagataaccagc |  |
| TgoT_E664VMA | gggtgatctgTKBatagataaccagc |  |
| TgoT_E664ATG | gggtgatctgCATatagataaccagc |  |
| TgoT_E664TGG | gggtgatctgCCAatagataaccagc |  |
| TgoT_G711NDT | ggaattgcgcggtcAHNaatgcgaccactg |  |
| TgoT_G711VMA | ggaattgcgcggtcTKBaatgcgaccactg |  |
| TgoT_G711ATG | ggaattgcgcggtcCATaatgcgaccactg |  |
| TgoT_G711TGG | ggaattgcgcggtcCCAaatgcgaccactg |  |
| TgoT_N735NDT | cggcagaacctgAHNttcaatgtaatactcgg |  |
| TgoT_N735VMA | cggcagaacctgTKBttcaatgtaatactcgg |  |
| TgoT_N735ATG | cggcagaacctgCATttcaatgtaatactcgg |  |
| TgoT_N735TGG | cggcagaacctgCCAttcaatgtaatactcgg |  |
| TgoT_block | ctttgaagccaaatttttc | Prevent aberrant annealing by pET_DA_as_padlock |
| pET29a_SphIcut | CTCCTTGCATGCACCATT | Provide double stranded region for pET29a cleavage by SphI. |
|  | | |
| Inner oligonucleotides (T7RSS) | | |
| T7RSS_R96/K98NS | GAAGTGAAAGCAAAAnnsGGCnnsCGGCCTACAGC | T7RSS library generation. Encoded |
| T7RSS_E207NNS | GTTGGCATAAAnnsGACAGTATAC |  |
| T7RSS_E222NNS | GAGATGTTGATCnnsAGTACGGGAATG |  |
| T7RSS_N748NNS | CAAACAAGACTTnnsCTAATGTTC |  |
| T7RSS_P759NNS | GTTTAGACTACAAnnsACCATAAACAC |  |
|  |  |  |
| Inner oligonucleotides (KOD) | | |
| KOD_Leu75CTA | gaagaagttcctAgggagaccag | Silent mutations to remove BsaI sites from KOD DNA polymerase gene to facilitate type IIS cloning. |
| KOD_Leu463CTA | cgagcctgctAggagacctcc |  |
| KOD_V93E | ccgcaggacgAAccagcgataag | Mutagenic oligonucleotides to graft mutations from polymerase 6G12 (evolved in Tgo DNA polymerase (22)) onto KOD DNA polymerase. Mutations capitalized. |
| KOD_A485L | caggcagaggTTGatcaagatcc |  |
| KOD_V589A | ggcttcttcgCtacgaagaagaag |  |
| KOD_609K610M | gcgcggacttAAAatGgtgaggcgtg |  |
| KOD_10A_6G12 | ggttccgccggagCAActggtgatccacCaAcCTataacgaAACaAttaCaTgactacCGTgcaCGTggtccccacgttAGTgttgccaagagg |  |
| KOD_704P | CtacatcgtgcCtaagggctctg |  |
| KOD_730G | GtacgacgccgGTtactacattg |  |
|  | | |
| Boundary oligonucleotides | | |
| CAT_L45CTG | CCCCTTATTAGCGTTTGCCAGGTCTCc'ggcc,tttCTGaagaccgtaaagaaaaataagc | Encode a mutation, prime extension in isothermal assembly in CAT and provide outnesting PCR tag. |
| CAT_L205/208CTGv2 | ggcagaatgCTGaatgaaCTGcaacagtactgc’GATG,AGAGACCCATCTGACCTCTGTGCTGCT/3invdT/ | Encode a mutation, serve as end point to isothermal assembly of CAT and encode outnest PCR tag. |
| pet29a_DA_as_theta | GTTAAACAAAATTATTTCTAGAGGGGAATTGTTA,TCCG'CgagaccTGGCAAACGCTAATAAGGGGttttttAGCAGCACAGAGGTCAGATGggtctcC'TTCC,TTTCGGGCTTTGTTAGCAGC | Theta oligonucleotide to serve as both primer and termination point of TgoT assembly. |
| b.pBAD30_DA_F_BsaI | 5/biotin-TEG-CCCCTTATTAGCGTTTGCCAGGTCTCT'TTGG,GCTAACAGGAGGAATTAACC | Primes assembly on pBAD30 (for T7RSS assembly). 5’ biotin for recovery of nascent strand via streptavidin capture. |
| pBAD30_DA_R_BsaI | GCTCGAGATCTGCAGCTGGTAC'CATA,TGAGACCCATCTGACCTCTGTGCTGCT/invdT/ | End point of isothermal assembly on pBAD30 (for T7RSS). |
| pGDR11_DA_F_BsaI | /5bioTEG/CCCCTTATTAGCGTTTGCCAGGTCTCACTATGAGAGGATCTCACCATCAC | Primes assembly on pGDR11 (for KOD assembly). 5’ biotin for recovery of nascent strand via streptavidin capture. |
| pGDR11_DA_R_BsaI | CCTGCAGCCAAGCTTAATTAGCTGGAGACCCATCTGACCTCTGTGCTGCT/invdT/ | End point of isothermal assembly on pGDR11 (for KOD). |
|  |  |  |
| Miseq library oligonucleotides | | |
| P5_TgoT_378F | AATGATACGGCGACCACCGAGATCTACACTCTTTCCCTACACGACGCTCTTCCGATCTNNNGGCTACACCTGACGAACGTGAATTAG | Append index, P5 and P3 sequences to region of TgoT covering Tyr384 and Val389. |
| P3_TgoT_407R | CAAGCAGAAGACGGCATACGAGATCGGTCTCGGCATTCCTGCTGAACCGCTCTTCCGATCTGGTAATGATAATGCTAGGGTATAAG |  |
| P5_TgoT_482F | AATGATACGGCGACCACCGAGATCTACACTCTTTCCCTACACGACGCTCTTCCGATCTNNNCTTGTAAGAAGCTGCTGGATTACC | Append index, P5 and P3 sequences to region of TgoT covering Ala490 and Phe493. |
| P3_TgoT_513R | CAAGCAGAAGACGGCATACGAGATCGGTCTCGGCATTCCTGCTGAACCGCTCTTCCGATCTGTCGTCTCGATATACTGACG |  |
| P5_TgoT_580F | AATGATACGGCGACCACCGAGATCTACACTCTTTCCCTACACGACGCTCTTCCGATCTNNNAGTCAACTGCTGGAACTGGAATATGA | Append index, P5 and P3 sequences to region of TgoT covering Phe587. |
| P3_TgoT_618R | CAAGCAGAAGACGGCATACGAGATCGGTCTCGGCATTCCTGCTGAACCGCTCTTCCGATCTGCCTGTGTCTCCTTTGC |  |
| P5_TgoT_647F | AATGATACGGCGACCACCGAGATCTACACTCTTTCCCTACACGACGCTCTTCCGATCTNNNAGTTCCTTCGTATCGTGAAAGAGGTG | Append index, P5 and P3 sequences to region of TgoT covering Glu664. |
| P3_TgoT_678R | CAAGCAGAAGACGGCATACGAGATCGGTCTCGGCATTCCTGCTGAACCGCTCTTCCGATCTCTTGGCAACTGCAACATG |  |
| P5_TgoT_705F | AATGATACGGCGACCACCGAGATCTACACTCTTTCCCTACACGACGCTCTTCCGATCTNNNATGTCAGCTACATCGTGCTGAAAG | Append index, P5 and P3 sequences to region of TgoT covering Gly711 and Asn735. |
| P3_TgoT_740R | CAAGCAGAAGACGGCATACGAGATCGGTCTCGGCATTCCTGCTGAACCGCTCTTCCGATCTGCGTAAAATGCGTTCCAC |  |
| P5_16_CAT_491F | AATGATACGGCGACCACCGAGATCTACACTCTTTCCCTACACGACGCTCTTCCGATCTNNNCCGTCCggtgagctggtgatatgggatagtg | Append index, P5 and P3 sequences to region of CAT covering Ser102 – Leu105 for sequencing of alanine scanning and insertion/deletion experiments. There are 2 forward primers as 2 different barcodes were used. |
| P5_17_CAT_491F | AATGATACGGCGACCACCGAGATCTACACTCTTTCCCTACACGACGCTCTTCCGATCTNNNGTAGAGggtgagctggtgatatgggatagtg |  |
| P3_CAT_606R | CAAGCAGAAGACGGCATACGAGATCGGTCTCGGCATTCCTGCTGAACCGCTCTTCCGATCTgaaataggccaggttttcaccgtaac |  |

**TABLE S2: Observed codon frequencies for single NNS insertion between CAT Phe102 and Ser103 (22611 reads).**

| **Codon** | **% expected** | **% observed** |
| --- | --- | --- |
| AAA | 5.0 | 1.86 |
| AAT | 5.0 | 1.88 |
| ACA | 5.0 | 1.23 |
| AGT | 5.0 | 4.47 |
| ATG | 5.0 | 2.19 |
| ATT | 5.0 | 3.49 |
| CAA | 5.0 | 0.70 |
| CAT | 5.0 | 3.88 |
| CCA | 5.0 | 2.45 |
| CGT | 5.0 | 0.99 |
| CTT | 5.0 | 1.63 |
| GAA | 5.0 | 1.71 |
| GAT | 5.0 | 1.97 |
| GCA | 5.0 | 1.62 |
| GGT | 5.0 | 1.98 |
| GTT | 5.0 | 2.71 |
| TAT | 5.0 | 3.86 |
| TGG | 5.0 | 3.75 |
| TGT | 5.0 | 3.39 |
| TTT | 5.0 | 2.84 |

**TABLE S3: Observed codon frequencies for double NNS insertion between CAT Phe102 and Ser103** **(22611 reads).**

| **Codon** | **% expected** | **% observed Insertion 1** | **% observed Insertion 2** |
| --- | --- | --- | --- |
| AAA | 5.0 | 3.15 | 2.87 |
| AAT | 5.0 | 5.46 | 3.19 |
| ACA | 5.0 | 1.78 | 0.99 |
| AGT | 5.0 | 4.74 | 1.76 |
| ATG | 5.0 | 3.21 | 1.59 |
| ATT | 5.0 | 5.40 | 4.91 |
| CAA | 5.0 | 1.09 | 1.37 |
| CAT | 5.0 | 3.41 | 2.34 |
| CCA | 5.0 | 1.71 | 2.49 |
| CGT | 5.0 | 1.59 | 1.31 |
| CTT | 5.0 | 2.81 | 1.46 |
| GAA | 5.0 | 2.01 | 2.17 |
| GAT | 5.0 | 1.01 | 3.66 |
| GCA | 5.0 | 4.29 | 2.36 |
| GGT | 5.0 | 2.12 | 1.67 |
| GTT | 5.0 | 1.76 | 1.48 |
| TAT | 5.0 | 2.96 | 2.21 |
| TGG | 5.0 | 5.31 | 2.66 |
| TGT | 5.0 | 4.41 | 2.40 |
| TTT | 5.0 | 3.32 | 2.83 |
| ACT | - | 0.15 | - |

**TABLE S4: Relative CAT deletion frequencies.** These are fractions of the sequences containing a deletion (21915 reads). The proportion of the whole dataset with deletions cannot be calculated because of overlapping Miseq barcodes.

|  | **Phe102** | **Ser104** | **Phe102+Ser104** |
| --- | --- | --- | --- |
| **Expected** | 0.33 | 0.33 | 0.33 |
| **Observed** | 0.48 | 0.483 | 0.037 |

**TABLE S5: Predicted impact on mutagenic primer melting temperature due to deletions.** CAT deletion oligonucleotide melting temperatures, calculated using MELTING 5 (http://www.ebi.ac.uk/biomodels/tools/melting/melt.php ) with potassium = 50 mM, magnesium = 10 mM, TRIS = 20 mM, nucleic acid concentration in excess 10 µM and all other values default.

| **Oligonucleotide** | **Calculated melting temperature (˚C)** |
| --- | --- |
| CAT_L105CTG2 | 76.3 |
| CAT_F102del,L105CTG | 67.8 |
| CAT_S104del,L105CTG | 67.2 |
| CAT_F102del,S104del,L105CTG | 61.0 |

**TABLE S6: T7RSS library generation Miseq data.** All values are expressed as percentages (given to 2 decimal places) of total reads. Only frequencies >0.1% are reported. Wild type codons are highlighted in pale grey. Read counts: 0.69x10^6^ (Glu96 / Lys98), 0.47x10^6^ (Glu207/ Glu222), 0.64x10^6^ (Asn748 / Pro759).

| **Codon** | **Expected** | **Glu96** | **Lys98** | **Glu207** | **Glu222** | **Asn748** | **Pro759** |
| --- | --- | --- | --- | --- | --- | --- | --- |
| AAC | 3.13 | 3.65 | 5.08 | 1.13 | 1.24 | 4.75 | 2.33 |
| AAG | 3.13 | 4.28 | 7.41 | 2.48 | 4.59 | 4.25 | 1.83 |
| ACC | 3.13 | 2.04 | 2.78 | 0.15 | 1.85 | 2.66 | 2.65 |
| ACG | 3.13 | 3.14 | 2.73 | 2.82 | 2.77 | 3.09 | 4.17 |
| AGC | 3.13 | 4.19 | 4.40 | 2.03 | 2.95 | 5.80 | 3.12 |
| AGG | 3.13 | 6.56 | 6.17 | 7.30 | 7.77 | 3.64 | 1.38 |
| ATC | 3.13 | 3.44 | 3.48 | 0.85 | 2.54 | 3.59 | 2.26 |
| ATG | 3.13 | 3.86 | 4.37 | 1.79 | 2.85 | 2.66 | 3.04 |
| CAC | 3.13 | 3.02 | 2.30 | 0.57 | 2.28 | 3.91 | 3.27 |
| CAG | 3.13 | 3.62 | 2.28 | 2.30 | 3.08 | 1.30 | 3.01 |
| CCC | 3.13 | 1.05 | 1.74 | 0.17 | 1.29 | 1.02 | 2.32 |
| CCG | 3.13 | 1.36 | 1.76 | 1.72 | 1.62 | 0.86 | 4.56 |
| CGC | 3.13 | 2.58 | 1.68 | 1.48 | 1.41 | 4.53 | 4.45 |
| CGG | 3.13 | 3.78 | 2.48 | 5.72 | 2.41 | 0.86 | 2.92 |
| CTC | 3.13 | 2.47 | 1.21 | 0.70 | 2.30 | 2.07 | 2.83 |
| CTG | 3.13 | 2.33 | 1.27 | 2.80 | 2.69 | 1.21 | 3.99 |
| GAC | 3.13 | 3.14 | 3.39 | 0.88 | 2.88 | 5.77 | 2.77 |
| GAG | 3.13 | 4.33 | 3.07 | 7.15 | 6.54 | 6.27 | 1.58 |
| GCC | 3.13 | 1.87 | 2.33 | 0.16 | 1.04 | 4.29 | 4.00 |
| GCG | 3.13 | 2.19 | 2.45 | 3.30 | 1.01 | 1.54 | 5.77 |
| GGC | 3.13 | 3.02 | 4.57 | 1.42 | 3.77 | 8.64 | 1.98 |
| GGG | 3.13 | 5.35 | 5.63 | 21.15 | 4.53 | 6.25 | 3.30 |
| GTC | 3.13 | 1.25 | 3.29 | 0.64 | 3.98 | 1.91 | 0.54 |
| GTG | 3.13 | 3.16 | 3.59 | 10.36 | 4.90 | 1.18 | 3.25 |
| TAC | 3.13 | 3.38 | 3.20 | 1.64 | 2.30 | 3.62 | 3.01 |
| TAG | 3.13 | 3.72 | 4.23 | 2.15 | 4.82 | 1.92 | 2.91 |
| TCC | 3.13 | 1.86 | 1.44 | 0.20 | 0.54 | 1.72 | 2.64 |
| TCG | 3.13 | 2.37 | 1.57 | 3.57 | 2.26 | 1.28 | 4.13 |
| TGC | 3.13 | 2.46 | 2.28 | 1.07 | 3.87 | 4.62 | 4.54 |
| TGG | 3.13 | 4.27 | 3.06 | 7.12 | 7.16 | 1.50 | 3.94 |
| TTC | 3.13 | 2.68 | 2.23 | 1.44 | 2.23 | 2.74 | 2.48 |
| TTG | 3.13 | 3.33 | 2.30 | 3.65 | 4.20 | 0.47 | 4.63 |
| AGA | - | 0.14 | - | - | - | - | - |
| AAA | - | - | 0.13 | - | - | - | - |
| GAA | - | - | - | - | 0.20 | - | - |
| CCA | - | - | - | - | - | - | 0.24 |

**TABLE S7: TgoT library generation Miseq data for 20 codon diversity.** All values are expressed as percentages (given to 2 decimal places) of total reads. Only frequencies >0.1% are reported. Wild-type codons are highlighted in pale grey. Read counts: 4.76x10^6^ (Val389), 0.61x10^6^ (Phe493), 2.60x10^6^ (Glu664), 2.61x10^6^ (Gly711/Asn735).

| **Codon** | **Expected** | **Val389** | **Phe493** | **Phe587** | **Glu664** | **Gly711** | **Asn735** |
| --- | --- | --- | --- | --- | --- | --- | --- |
| AAA | 5.0 | 4.00 | 3.28 | 2.27 | 2.94 | 5.47 | 9.56 |
| AAT | 5.0 | 6.00 | 3.19 | 3.35 | 7.38 | 7.34 | 11.35 |
| ACA | 5.0 | 5.60 | 4.51 | 5.97 | 3.91 | 7.89 | 5.04 |
| AGT | 5.0 | 5.10 | 4.51 | 3.47 | 3.83 | 7.10 | 5.48 |
| ATG | 5.0 | 7.60 | 6.65 | 9.02 | 6.50 | 4.49 | 5.72 |
| ATT | 5.0 | 4.80 | 8.51 | 7.11 | 3.69 | 4.59 | 6.77 |
| CAA | 5.0 | 5.20 | 4.93 | 1.95 | 4.15 | 3.49 | 4.13 |
| CAT | 5.0 | 6.10 | 5.72 | 4.56 | 6.83 | 4.49 | 7.48 |
| CCA | 5.0 | 5.70 | 3.19 | 6.83 | 5.02 | 3.22 | 2.05 |
| CGT | 5.0 | 4.50 | 2.85 | 1.22 | 3.95 | 5.81 | 3.61 |
| CTT | 5.0 | 4.90 | 2.56 | 4.23 | 3.56 | 4.32 | 4.64 |
| GAA | 5.0 | 3.90 | 4.64 | 1.29 | 4.74 | 5.83 | 3.39 |
| GAT | 5.0 | 5.40 | 6.24 | 5.54 | 8.64 | 6.79 | 6.65 |
| GCA | 5.0 | 7.50 | 4.65 | 2.10 | 3.96 | 2.59 | 3.41 |
| GGT | 5.0 | 2.10 | 3.00 | 2.97 | 3.70 | 3.94 | 22.84 |
| GTT | 5.0 | 3.50 | 6.44 | 6.86 | 4.14 | 4.40 | 3.76 |
| TAT | 5.0 | 3.70 | 8.21 | 7.55 | 4.80 | 5.11 | 3.40 |
| TGG | 5.0 | 6.00 | 7.37 | 5.98 | 8.22 | 4.60 | 3.26 |
| TGT | 5.0 | 3.80 | 4.44 | 10.28 | 3.66 | 4.46 | 3.50 |
| TTT | 5.0 | 2.30 | 5.03 | 5.50 | 3.34 | 3.12 | 3.19 |
| TTG | - | 0.10 | - | - | - | - | - |
| GTG | - | 1.60 | - | - | - | - | - |
| AAC | - | - | - | 0.10 | - | - | - |
| ACC | - | - | - | 0.22 | - | - | - |
| CCC | - | - | - | 0.27 | - | - | - |
| TTC | - | - | - | 0.66 | - | - | - |
| GAG | - | - | - | - | 1.96 | - | - |
| GGC | - | - | - | - | - | 0.32 | - |

**TABLE S8: TgoT library generation Miseq data for reduced diversity mutagenesis.** All values are expressed as percentages (given to 2 decimal places) of total reads. Only frequencies >0.1% are reported. Wild type codons are highlighted in pale grey. Read counts: 4.76x10^6^ (Tyr384), 0.61x10^6^ (Ala490).

Specific diversity data:

| **Tyr384 -> YWC** | | |
| --- | --- | --- |
| **Codon** | **Expected** | **Tyr384** |
| CAC | 25 | 19.80 |
| CTC | 25 | 19.00 |
| TAC | 25 | 35.10 |
| TTC | 25 | 24.20 |
| TCT | - | 1.30 |
| **Ala490 -> SRC** | | |
| **Codon** | **Expected** | **Ala490** |
| CAC | 25 | 17.29 |
| CGC | 25 | 24.16 |
| GAC | 25 | 28.16 |
| GGC | 25 | 29.70 |
| GCA | - | 0.40 |

**Supplementary Protocol 1: T7RSS library using biotinylated oligonucleotide and streptavidin cleanup, and critical experimental considerations**

For T7RSS (and KOD DNA polymerase) we used 2 boundary oligonucleotides with a 5’ biotin on the oligonucleotide that initiates (primes) the isothermal assembly reaction. This allowed specific recovery of full length products using paramagnetic streptavidin-coated beads and removal of any partial synthesis products or unextended oligonucleotides by sodium hydroxide washing. For T7RSS, we also inserted 2 additional BspQI sites in the plasmid near the 3’ of the T7RP gene, as we had observed that the sole BspQI site on the plasmid was to the 5’ of the gene, meaning ~7.5 kb (almost the entire plasmid) had to be degraded by exonuclease III. This had a significant impact on the results, suggesting the exonuclease III step required optimisation from our original condition (as indeed the NEB manual states).

1) Generate ssDNA by nicking restriction endonuclease and exonuclease III digest. The below reaction was made up in a 0.2 ml tube and incubated 2 h 37˚C, 20 min 80˚C.

|  | Stock concentration | Reaction concentration | Volume |
| --- | --- | --- | --- |
| Plasmid pBAD30b2_T7RSS-DA | 0.19 µM (915 ng/µl) | 0.06 µM (283.6 ng/µl) | 9.3 µl |
| NEB3.1 buffer | 10x | 1x | 2 µl |
| Nt.BspQI | 5U/µl | 3U / µg DNA | 2.6 µl |
| Exonuclease III | 100U/µl | 40U / µg DNA | 3.4 µl |
| Water |  |  | 11.7 µl |

2) Phosphorylate oligonucleotides.

Inner oligonucleotides were phosphorylated separately from the boundary oligonucleotide.

The below reactions were made up in a 0.2 ml tube and incubated 2 h at 37˚C, followed by 20 min at 80˚C. T4 PNK was used in Cutsmart supplemented with ATP to ensure buffer compatibility.

**Inner oligonucleotides:**

| Component | Stock concentration | Reaction concentration | Volume |
| --- | --- | --- | --- |
| T7RSS_R96/K98NNS | 100 µM | 17 µM | 8.5 µl |
| T7RSS_E207NNS | 100 µM | 17 µM | 8.5 µl |
| T7RSS_E222NNS | 100 µM | 17 µM | 8.5 µl |
| T7RSS_N748NNS | 100 µM | 17 µM | 8.5 µl |
| T7RSS_P759NNS | 100 µM | 17 µM | 8.5 µl |
| Cutsmart buffer | 10 x | 1 x | 5 µl |
| ATP | 100 mM | 1 mM | 0.5 µl |
| T4 PNK | 10 U/µl | 0.5 U/µl | 1 µl ( |
| Water |  |  | 1 µl |

**Boundary oligonucleotide:**

The 5’ oligonucleotide (b.pBAD30_DA_F_BsaI) does not need to be and cannot be phosphorylated (5’ is blocked by the biotin moiety).

| Component | Stock concentration | Reaction concentration | Volume |
| --- | --- | --- | --- |
| pBAD30_DA_R_BsaI | 100 µM | 10 µM | 1 µl |
| Cutsmart buffer | 10 x | 1 x | 1 µl |
| ATP | 10 mM | 1 mM | 1 µl |
| T4 PNK | 10 U/µl | 0.5 U/µl | 0.2 µl |
| Water |  |  | 6.8 µl |

3) Prepare for Darwin Assembly reaction

Both single stranded plasmid DNA and phosphorylated oligonucleotides were used with no purification beyond heat inactivation of the enzymes.

In a 0.2 ml tube, combine:

- 4 µl single stranded plasmid DNA (0.24 pmol)
- 1.17 µl boundary oligonucleotides (mixed at 2.05 µM each; 2.4 pmol each)
- 2.82 µl inner oligonucleotides (48 pmol each inner oligonucleotide)

Freeze for >10 min and return to RT or heat anneal (5 min 95˚C and cool to 4˚C at 0.1˚C/sec).

This gives a 10-fold excess of boundary oligonucleotides over plasmid and a 200-fold excess of each inner oligonucleotide over plasmid.

4) Carry out Darwin Assembly isothermal assembly reaction

Add 1 volume 2x Darwin Assembly mix to the annealed DNA (in this case, 8.03 µl) and incubate for 1 h at 50˚C. Reaction time was based on the expected performance of DNA polymerases in PCR conditions and thus probably unnecessary long. A 10 min reaction is sufficient for a T7RP assembly with 5 point mutations (data not shown).

5) Streptavidin cleanup

5 µl Dynabeads MyOne Streptavidin C1 beads had been blocked in 2x BWBS-T for over an hour. After the assembly reaction, the reaction volume was adjusted to 50 µl with water (34 µl added). The beads were suspended in 50 µl 2x BWBS-T and added directly to the assembly reaction, and transferred to a 1.5 ml tube. This was incubated for 3 h at room temperature and the beads then washed on a magnetic stand:

- wash 1: 200 µl (37˚C) 30 mM NaOH
- wash 2: 200 µl (37˚C) 30 mM NaOH
- wash 3: 200 µl EB-T (10 mM TRIS.HCl pH 8.8, 0.1 mM EDTA, 0.01% Tween-20)

After washing, the beads were suspended in 10 µl EB (10 mM TRIS.HCl pH 8.8) and used directly for PCR. We determined that 10 min capture is sufficient for a T7RP assembly with 5 point mutations (data not shown).

6) PCR amplify nascent strand

The cleaned-up assembly reaction can now be used directly as a PCR template:

Make up the following reaction mix:

|  | Stock solution | Final concentration | Volume |
| --- | --- | --- | --- |
| KOD Xtreme buffer | 2 x | 1 x | 40 µl |
| Primer outnest1 / outnest2 pre-mix | 50 µM each | 0.3 µM each | 0.48 µl |
| dNTPs | 2 mM each | 0.4 mM each | 16 µl |
| KOD Xtreme DNA polymerase | 1 U/µl | 0.01 U/µl | 0.8 µl |
| Water |  |  | 19.52 µl |

We split to 3 x 24 µl and added 1 µl beads, 1 µl beads diluted 1/10 in EB or just EB. Thermocycling conditions:

- 2 min 95 ˚C
- 28 cycles of: 15 sec 98˚C, 30 sec 64˚C, 2 min 30 s 68˚C
- 6 min 72˚C

7) Clone PCR product

After a pilot PCR to confirm assembly, PCR is scaled up to generate non-limiting material for efficient cloning (using Monarch DNA Gel Extraction Kit from NEB). BsaI sites encoded in the original boundary oligonucleotides allow removal of outnest priming sites and prepare the insert for cloning. A complementary vector was made using Q5 HS DNA polymerase and primers pBAD30_DA_vec_F2 and pBAD30_DA_vec_R2 from the parental pBAD30b2_T7RSS plasmid and also BsaI digested.

For library generation, we ligated 75 fmol vector and 300 fmol insert in 100 µl in 1x T4 DNA ligase buffer (NEB), with 40U T4 DNA ligase and 50U 5’deadenylase. The ligation was incubated at room temperature phenol:chloroform extracted, isopropanol precipitated and transformed into home-made NEB 10β electrocompetent cells (from a 200 ml culture). We obtained 1x10^7^ colonies.

**Critical experimental parameters**

There are few points in Darwin Assembly that tend to be the source of experimental problems. In general, we have found it to be a robust method. Failures tend to be a result of ineffective ssDNA generation (1) or PCR (2). We have also included a brief discussion on reducing library bias (3).

1. **ssDNA generation**

In principle, any nicking restriction endonuclease can be used, provided it has good activity at 37˚C in a buffer compatible with exonuclease III (we have used both NEB 3.1 and Cutsmart successfully and have not tested other buffers).

1. We suggest ensuring ssDNA is being generated effectively the first time a new plasmid is tested, or when a PCR fails. We do this by running a timecourse of nicking/exoIII reaction (e.g. take samples at 0, 30, 60, 120 min), running an unstained agarose gel and post-staining with SYBR Gold (or other stain that stains ssDNA effectively). From this it should evident that the plasmid migrates faster as the targeted strand is degraded.
2. Choice of nicking endonuclease. Mostly we have used Nt.BspQI, but only for reasons of convenience. We have had problems using Nt.BbvCI, possibly due to the high glycerol concentrations in our reactions. While it is likely that less enzyme could be used, this would require some titration and we instead inserted an Nt.BspQI site by iPCR.
3. **PCR failure.**

Failed PCRs usually have either of two outcomes: too many amplicons or no amplified product. We routinely gel purify PCR products to be used for cloning (even when no secondary bands are visible using SYBR safe staining and blue light excitation). Purification substantially improves the number of clones with full length products cloned.

- 1. Multiple bands running shorter than the desired product. This is likely caused by carried over inner oligonucleotides, which poison the outnested PCR. In this case, the amount of inner oligonucleotide carried over needs to be reduced. This can sometimes be achieved by reducing the inner oligo:plasmid ratio (e.g. reduce excess from 250:1 to 50:1) or by diluting the Darwin Assembly reaction prior to PCR (e.g. 1/10). We have noticed that assembly strategy (biotinylated boundaries or theta) can affect PCR efficiency.
  2. No PCR bands. We rarely had this problem. The PCR should light up in relatively few (22 – 28) cycles. Full length bands obtained from reactions carried out for more than 32 cycles were generally background (all clones wild-type). Generally, the easiest solution was to switch PCR enzyme (e.g. from Q5 Hot Start DNAP to KOD Xtreme DNAP). We usually use Q5 Hot Start DNAP for cloning for its high fidelity, but KOD Xtreme proved most successful and became our first choice.

1. **Library bias.**

It is clear from our deep sequencing data that there are biases to the library content, especially with regards to the InDel library. We have not optimised this, as our primary interest was ensuring all possible mutations were represented at every position.

Our data suggest that the main cause of biases is the expected variation on mutagenic primer melting temperatures. Thus, if evenly weighted libraries are a priority, we recommend mutagenic oligos to be designed to have melting temperatures as homogeneous as possible.

**Supplementary protocol 2: Tgo library using theta oligonucleotide and exonuclease cleanup**

The Tgo (*Thermococcus gorgonarius* DNA polymerase) library was generated using a theta oligonucleotide to enable the selective degradation of partial assembled reactions and unextended oligos. The theta oligonucleotide is essentially the 5’ and 3’ boundary oligonucleotides of an assembly reaction joined together and separated by a flexible linker (poly-dT), akin to a padlock probe. This means that successful assembly generates a closed circle and any partial synthesis products, excess primers and other contaminants can be removed by exonuclease I and exonuclease III digestion.

1) Generate ssDNA by nicking restriction endonuclease and exonuclease III digest.

The below reaction was made up in a 0.2 ml tube and incubated 2 h 37˚C, 20 min 80˚C.

|  | Stock concentration | Reaction concentration | Volume |
| --- | --- | --- | --- |
| Plasmid pET29aΔΔΔ_TgoT(I521L) | 0.1 µM (428 ng/µl) | 0.06 µM (264.5 ng/µl) | 12.4 µl |
| NEB3.1 buffer | 10x | 1x | 2 µl |
| Nt.BspQI | 5U/µl | 3U / µg DNA | 1.6 µl |
| Exonuclease III | 100U/µl | 40U / µg DNA | 2.1 µl |
| Water |  |  | 0.5 µl |

2) Phosphorylate oligonucleotides.

Here, the inner oligonucleotides were phosphorylated separately from the theta oligonucleotide to allow versatility during optimisation. All inner oligonucleotides here included at least one codon diversified to all 20 codons to make small-intelligent libraries (34). This involves ordering 4 oligonucleotides per targeted codon with NDT, VMA, ATG and TGG codons and mixing at 12:6:1:1 to give (theoretically) a balanced mixture of 20 codons, encoding all 20 amino acids. Oligonucleotide concentrations given here assume these 4 have been mixed at 12:6:1:1, indicated by “X”. The other oligonucleotides are named according to the nucleotides they encode, e.g. TgoT_384YWC encodes the degenerate codon YWC at amino acid 384, encoding CAC, TAC, CTC and TTC.

The below reactions were made up in a 0.2 ml tube and incubated 2 h 37˚C, 20 min 80˚C. T4 PNK was used in Cutsmart supplemented with ATP to ensure buffer compatibility.

**Inner oligonucleotides:**

| Component | Stock concentration | Reaction concentration | Volume |
| --- | --- | --- | --- |
| TgoT_384YWC, 389X | 100 µM | 14 µM | 7 µl |
| TgoT_490RSC, 493X | 100 µM | 14 µM | 7 µl |
| TgoT_F587X | 100 µM | 14 µM | 7 µl |
| TgoT_E664X | 100 µM | 14 µM | 7 µl |
| TgoT_G711X | 100 µM | 14 µM | 7 µl |
| TgoT_N375X | 100 µM | 14 µM | 7 µl |
| TgoT_block | 100 µM | 3 µM | 1.5 µl |
| Cutsmart buffer | 10 x | 1 x | 5 µl |
| ATP | 100 mM | 1 mM | 0.5 µl |
| T4 PNK | 10 U/µl | 0.5 U/µl | 1 µl |
| Water |  |  | 0 µl |

**Theta oligonucleotide:**

| Component | Stock concentration | Reaction concentration | Volume |
| --- | --- | --- | --- |
| pET29a_DA_as_theta | 100 µM | 10 µM | 5 µl |
| Cutsmart buffer | 10 x | 1 x | 5 µl |
| ATP | 10 mM | 1 mM | 5 µl |
| T4 PNK | 10 U/µl | 0.5 U/µl | 1 µl |
| Water |  |  | 38.5 µl |

3) Prepare for Darwin Assembly reaction

Both single stranded plasmid DNA and phosphorylated oligonucleotides were used with no purification beyond heat inactivation of the enzymes.

In a 0.2 ml tube, combine:

- 4 µl single stranded plasmid DNA (0.24 pmol)
- 2.4 µl 2.05 µM theta oligonucleotide (2.4 pmol)
- 3.43 µl inner oligonucleotides (48 pmol each inner oligonucleotide)

Freeze for >10 min and return to RT.

This gives a 10-fold excess of theta oligonucleotide over plasmid and a 200-fold excess of each inner oligonucleotide over plasmid.

4) Carry out Darwin Assembly isothermal assembly reaction

Add 1 volume 2x Darwin Assembly mix to the annealed DNA (in this case, 8 µl) and incubate for 1 h at 50˚C.

5) Exonuclease cleanup

A 5x mastermix of exonuclease I (to degrade single stranded DNA), exonuclease III (to degrade one strand of linear dsDNA), a targeting oligonucleotide that hybridises to the plasmid to generate a double stranded restriction enzyme recognition site and an appropriate restriction endonuclease was then added. This mix ought to linearize the plasmid and efficiently degrade any double- or single-stranded DNA with free ends.

|  | Stock solution | 5x concentration | 1x concentration | Volume for 5x mastermix |
| --- | --- | --- | --- | --- |
| Exonuclease I (U/µl) | 20 | 4 | 0.8 | 2 µl |
| Exonuclease III (U/µl) | 10 | 2 | 0.4 | 2 µl |
| SphI-HF(U/µl) | 10 | 1 | 0.2 | 1 µl |
| pET_SphI_cut (µM) | 100 | 50 | 10 | 5 µl |

4 µl of 5x mastermix were added to the 16 µl DA reaction and incubated 40 m 37˚C, 20 m 80˚C.

6) PCR amplify nascent strand

The cleaned-up assembly reaction can now be used directly as a PCR template:

Make up the following reaction mix:

|  | Stock solution | Final concentration | Volume |
| --- | --- | --- | --- |
| Q5 buffer | 5 x | 1 x | 12 µl |
| CES | 5 x | 1 x | 12 µl |
| Primer outnest1 / outnest2 pre-mix | 50 µM each | 0.5 µM each | 0.6 µl |
| dNTPs | 10 mM each | 0.2 mM each | 1.2 µl |
| Q5 HS DNA polymerase | 2U/µl | 0.01U/µl | 0.3 µl |
| Water |  |  | 31.5 µl |

Split to 2 x 24 µl and add 1 µl Darwin Assembly reaction to one (the other serves as the no template PCR). Thermocycle:

- 1 min 98 ˚C
- 25 cycles of: 15 sec 98˚C, 2 min 72˚C
- 1 min 72˚C

While Q5 did amplify this product, we actually cloned using MyTaq HS DNA polymerase as the resulting amplicon looked better resolved in the agarose gel:

|  | Stock solution | Final concentration | Volume |
| --- | --- | --- | --- |
| MyTaq buffer | 5 x | 1 x | 12 µl |
| Primer outnest1 / outnest2 pre-mix | 50 µM each | 0.4 µM each | 0.48 µl |
| Q5 HS DNA polymerase | 2 U/µl | 0.01 U/µl | 0.3 µl |
| Water |  |  | 44.82 µl |

Split to 2 x 24 µl and add 1 µl Darwin Assembly reaction to one (the other serves as the no template PCR). Thermocycle:

- 1 min 98 ˚C
- 25 cycles of: 15 sec 98˚C, 15 sec 55˚C, 1 min 72˚C
- 1 min 72˚C

7) Clone PCR product

After a pilot PCR, this was scaled up and the band gel purified to improve cloning efficiency (using Monarch DNA Gel Extraction Kit from NEB). We encoded two BsaI sites in the theta oligonucleotide such that a BsaI digest cut the PCR product at both ends to prepare the insert for cloning. A complementary vector was made using Q5 HS DNA polymerase and primers pET_assembly_vec_F and pET_assembly_pET_vec_R from the parental pET29aΔΔΔ_TgoT and also BsaI digested. For library generation, we ligated 187.5 fmol vector and 750 fmol insert in 100 µl in 1x T4 DNA ligase buffer (NEB), with 40U T4 DNA ligase and 50U 5’deadenylase. The ligation was incubated at room temperature for 1 h and 40 min. DNA was phenol:chloroform extracted, isopropanol precipitated and transformed into home-made NEB 10β electrocompetent cells (from a 200 ml culture). We obtained 2.25x10^8^ colonies.

**Supplementary protocol 4: codon frequency counting**

This is an outline of the methods used to calculate the frequency of each codon at each position from raw Illumina Miseq data. We begin by explaining our Miseq library design to make some of the steps clearer and then outline the method used. This is a method developed by molecular biologists for molecular biologists: programming is not required!

All software used is free and (with the exception of R) was used in Terminal in Mac OS X, although can also be run in Windows (e.g. using Cygwin) or Linux. The final step was carried out in Microsoft Excel.

Software required:

FASTX Toolkit: <http://hannonlab.cshl.edu/fastx_toolkit/index.html)>

TAGcleaner: <http://tagcleaner.sourceforge.net/>

RStudio: <https://www.rstudio.com/>

Awk (simple programming language transforming text, built into OS X)

Sed (Unix utility for transforming text, built into OS X)

**Miseq library design**

All the primers used to generate Miseq libraries have common features, meaning the libraries all share the design: 5’-P5_NNN_index_gene_P3-3’.

The Miseq libraries were prepared by PCR, using 2 long primers. This allowed targeting to a specific region of the gene and was the simplest way to append sequences necessary for Miseq sequencing. Note that the Miseq expects the barcode to be at the 3’ (between P3 and the gene sequence) so libraries designed this way have to be manually demultiplexed as described below.

P5: Sequence captured by the flow cell. Also encodes read 1 primer.

NNN: Random diversity. This library design can cause Miseq runs to fail as the initially sequenced bases are used by the Miseq for quality control (actually cluster identification) and very low diversity can lead to reads being lost or run failure.

Index: Barcode/index is a 6 nt sequence that allows unique identification of each read. A full list of barcodes is here: <https://wikis.utexas.edu/display/GSAF/Illumina+-+all+flavors>

Gene: Gene sequence specified by primer design.

P3: Read 2 primer (only needed for paired end reads but included as standard anyway).

**Data processing**

1. Trim FASTQ

Initially, the FASTQ files are trimmed to remove the initial NNN and to reduce the size. Full-length reads of 150 nucleotides are not required and quality declines as the read length gets longer. Hence, this prevents including unnecessary data in the quality filtering, which would waste reads that are high quality in the region we are concerned with:

*…bin/fastx_trimmer -Q 33 -f 4 -l 125 -i raw.fastq -o illumina/trim.fastq*

fastx_trimmer calls the FASTX Trimmer command

-Q 33 corrects the quality scoring to the correct Illumina Miseq version

-f 4 first base kept is number 4 (meaning the first 3 are trimmed off)

-l 125 last base kept is number 125.

-i input file

-o output file

1. Quality filter the trimmed FASTQ

This command ensures at least 90% of the base calls in each read are Q30 or higher, meaning the Miseq is 99.9% certain the base call is correct. See Illumina tech note for more information: <https://www.illumina.com/documents/products/technotes/technote_Q-Scores.pdf>

*…/fastq_quality_filter -Q 33 -q 30 -p 90 -v -i trim.fastq -o q30_trim.fastq*

fastq_quality_filter calls the FASTX Quality Filter command

-Q 33 corrects the quality scoring to the correct Illumina Miseq version

-q 30 quality threshold Q30

-p 90 minimum percentage of bases in each read that must reach quality threshold

-i input file

-o output file

example output:

Quality cut-off: 30

Minimum percentage: 90

Input: 21121540 reads.

Output: 18773973 reads.

discarded 2347567 (11%) low-quality reads.

1. Demultiplex

Now the FASTQ is trimmed to size and lower quality reads are removed, the file is split into multiple files according to the barcodes/indices encoded in each sample. The output from this script is FASTA.

*…cat q30_trim.fastq | …/fastx_barcode_splitter.pl --bol --exact --bcfile names.txt --prefix split/ --suffix.fa*

cat UNIX command to read a file, in this case the quality filtered, trimmed FASTQ.

| UNIX pipe. Passes contents of previous command to next one, so rather than opening the entire input file specified by cat, the file is instead used as input for the subsequent command.

Fastx_barcode_Splitter calls FASTX barcode splitter tool

--bol specifies barcode will be at “beginning of line” – the 5’ of the read

--exact barcode in read must exactly match the ones specified in the input file

--bcfile the file containing the barcodes and desired filenames, formatted as plain text and tab delimited, e.g.:

sample1 GGCTAC

sample2 CTTGTA

--prefix filename prefix.

--suffix filename extension.

The filename is determined by the combination of barcode file, prefix and suffix. Here for example, the prefix “split/” will put the files in a folder called ”split”, the files will be named “sample1” and “sample2”, as determined by the barcode file (names.txt) and the files will be .fq files, as specified by –suffix.

1. Trim each file.

The files are now trimmed again to remove the barcodes, as in step 1. This time however, “-f 7” is used so that the first 6 nucleotides (assuming a 6 nt barcode) is trimmed off. This must be repeated for each trimmed file.

1. Trim exact 3’ (optional)

For some datasets it is helpful to trim the file to an exact 3’ sequence. I didn’t generally do this, but it is helpful for variable length samples (the indel libraries) to generate exact sized files:

*perl tagcleaner-standalone-0.16/tagcleaner.pl -fasta infile.fa -out filename -out_format 1  -tag3 GAATACCACG —tag5 ACTGAA -nomatch 3 -verbose -minlen 27*

perl tagcleaner-standalone-0.16/tagcleaner.pl runs the TAGcleaner PERL script

-fasta: specifies input file is FASTA (can also use -fastq), followed by the input filename

-out: name of output file (here, “filename”)

-out_format: specifies out file format (1 = fasta, 3 = fastq)

-tag3: tag to search for at 3’ (cuts off anything after it)

-tag5: tag to search for at 5’ (does not cut off)

-nomatch 3: sequences deleted if either tag does not match (1= 5’ only, 2 = 3’ tag only,
4 = match either 5’ or 3’)

-verbose: report printed in Terminal

-minlen 27: sequences shorter than 27 nt deleted after tag trimming

1. Correct file spacing

The FASTQ files are now trimmed, quality filtered, de-multiplexed and converted to FASTA, and are ready for the codons to be counted. In order to simplify the reading of the data into R, we carried out some further text file manipulation in Terminal.

1. Firstly, we used awk to read every second line into a new file:

*awk '!(NR % 2)' reads.fa > line_reads.fa*

This removes the header lines from FASTA, e.g.

>seq1

GATCGATC

>seq2

TTCCGGAA

>seq3

AAACCGAA

Becomes:

GATCGATC

TTCCGGAA

AAACCGAA

1. If necessary, we corrected the file so that each sequence is in frame by removing 1 or 2 characters from the front of each line (that is, from the 5’ of each sequence):

*awk '{ print substr($0,2) }' codons_line_reads.fa > frame_codons_line_reads.fa*

In this case, 1 character is removed (the new file begins from the 2^nd^ character).

If it is necessary to remove 2 characters, use:

*awk '{ print substr($0,3) }' codons_line_reads.fa > frame_codons_line_reads.fa*

Note: the 2 in brackets has changed to a 3, to specify that each line in the new file begins from the 3^rd^ character in the original file.

1. Next, we used “sed” to insert a space every 3^rd^ character (in other words, to split into codons):

*sed 's/.\{3\}/& /g' line_reads.fa > codons_line_reads.fa*

1. Read into R.

The next few commands in RStudio will read the data into R, count the codons frequency at each position, calculate percentages and export the data as a CSV file ready to load into Excel. The below commands assume 5 codons are being counted to keep this document shorter. If more are use, simply repeat the commands being sure to add 1 number or letter each time, following the pattern in the first five.

Now that the data are processed, reading into R (using RStudio) is very simple. First set the working directory:

*setwd("/Users/chriscozens/illumina/“)*

And then read the data from the prepared text file into R (in this case, into a table named “data”):

*data = read.table("codons_line_reads.fa",header=FALSE)*

Each row should represent a unique sequence, as the sequence headers were removed using awk. Each column should be a codon because of the spaces inserted using the sed-command and the final awk-command should have ensured each codon is in frame.

1. The R package plyr is required for counting, so it is called:

*require(plyr)*

1. Count each column:

*count(nt[,1]) -> a*

*count(nt[,2]) -> b*

*count(nt[,3]) -> c*

*count(nt[,4]) -> d*

*count(nt[,5]) -> e*

1. Create column names:

*aname <- c("a", "afreq")*

*bname <- c("b", "bfreq")*

*cname <- c("c", "cfreq")*

*dname <- c("d", "dfreq")*

*ename <- c("e", "efreq")*

1. Append column names to columns:

*colnames(a) <- aname*

*colnames(b) <- bname*

*colnames(c) <- cname*

*colnames(d) <- dname*

*colnames(e) <- ename*

1. Sum each column

*sum(a[,2]) -> asum*

*sum(b[,2]) -> bsum*

*sum(c[,2]) -> csum*

*sum(d[,2]) -> dsum*

*sum(e[,2]) -> esum*

1. Calculate and append percentages

*cbind(a, (a[,2]/asum*100)) -> pc_a*

*cbind(b, (b[,2]/bsum*100)) -> pc_b*

*cbind(c, (c[,2]/csum*100)) -> pc_c*

*cbind(d, (d[,2]/dsum*100)) -> pc_d*

*cbind(e, (e[,2]/esum*100)) -> pc_e*

1. Create one table

*rbind.fill(pc_a,pc_b,pc_c,pc_d,pc_e,) -> pc_nt*

1. Export table as CSV file

*write.csv(pc_nt, file = "pc_nt.csv")*

The table pc_nt.csv (meaning percentage nucleotide) should now contain the percentage of each codon at each position and can be opened in Excel, or other spreadsheet software.
