# Supplementary material for: Darwin Assembly: fast, efficient, multi-site bespoke mutagenesis
Source: Nucleic Acids Res. 2018 Feb 2;46(8):e51. doi: 10.1093/nar/gky067 (PMC5934624; doi:10.1093/nar/gky067)
Supplement: Supplementary Data [file gky067_supp.zip › nar-03248-met-g-2017-File012.docx]

**pSB1C3A2**

cagaaatcatccttagcgaaagctaaggattttttttatctgaaattctgcctcgtgatacgcctatttttataggttaatgtcatgataataatggtttcttagacgtcaggtggcactcgagttgatcgggcacgtaagaggttccaactttcaccataatgaaataagatcactaccgggcgtattttttgagttatcgagattttcaggagctaaggaagctaaaatggagaaaaaaatcactggatataccaccgttgatatatcccaatggcatcgtaaagaacattttgaggcatttcagtcagttgctcaatgtacctataaccagaccgttcagctggatattacggcctttttaaagaccgtaaagaaaaataagcacaagttttatccggcctttattcacattcttgcccgcctgatgaatgctcatccggaatttcgtatggcaatgaaagacggtgagctggtgatatgggatagtgttcacccttgttacaccgttttccatgagcaaactgaaacgttttcatcgctctggagtgaataccacgacgatttccggcagtttctacacatatattcgcaagatgtggcgtgttacggtgaaaacctggcctatttccctaaagggtttattgagaatatgtttttcgtctcagccaatccctgggtgagtttcaccagttttgatttaaacgtggccaatatggacaacttcttcgcccccgttttcaccatgggcaaatattatacgcaaggcgacaaggtgctgatgccgctggcgattcaggttcatcatgccgtttgtgatggcttccatgtcggcagaatgcttaatgaattacaacagtactgcgatgagtggcagggcggggcgtaatttgatatcgagctcgcttggactcctgttgatagatccagtaatgacctcagaactccatctggatttgttcagaacgctcggttgccgccgggcgttttttattggtgagaatccaagcctcgagctgtcagaccaagtttactcatatatactttagattgatttaaaacttcatttttaatttaaaaggatctaggtgaagatcctttttgataatctcatgaccaaaatcccttaacgtgagttttcgttccactgagcgtcagaccccgtagaaaagatcaaaggatcttcttgagatcctttttttctgcgcgtaatctgctgcttgcaaacaaaaaaaccaccgctaccagcggtggtttgtttgccggatcaagagctaccaactctttttccgaaggtaactggcttcagcagagcgcagataccaaatactgttcttctagtgtagccgtagttaggccaccacttcaagaactctgtagcaccgcctacatacctcgctctgctaatcctgttaccagtggctgctgccagtggcgataagtcgtgtcttaccgggttggactcaagacgatagttaccggataaggcgcagcggtcgggctgaacggggggttcgtgcacacagcccagcttggagcgaacgacctacaccgaactgagatacctacagcgtgagctatgagaaagcgccacgcttcccgaagggagaaaggcggacaggtatccggtaagcggcagggtcggaacaggagagcgcacgagggagcttccagggggaaacgcctggtatctttatagtcctgtcgggtttcgccacctctgacttgagcgtcgatttttgtgatgctcgtcaggggggcggagcctgtggaaaaacgccagcaacgcggcctttttacggttcctggccttttgctggccttttgctcacatgttctttcctgcgttatcccctgattctgtggataaccgtattaccgcctttgagtgagctgataccgctcgccgcagccgaacgaccgagcgcagcgagtcagtgagcgaggaagcctgcataacgcgaagtaaCGCGGAACCCCTATTTGTTTATTTTTCTAAATACATTCAAATATGTATCCGCTCATGAGACAATAACCCTGATAAATGCTTCAATAATATTGAAAAAGGAAGAGTATGAGTATTCAACATTTCCGTGTCGCCCTTATTCCCTTTTTTGCGGCATTTTGCCTTCCTGTTTTTGCTCACCCAGAAACGCTGGTGAAAGTAAAAGATGCTGAAGATCAGTTGGGTGCACGAGTGGGTTACATCGAACTGGATCTCAACAGCGGTAAGATCCTTGAGAGTTTTCGCCCCGAAGAACGTTTTCCAATGATGAGCACTTTTAAAGTTCTGCTATGTGGCGCGGTATTATCCCGTGTTGACGCCGGGCAAGAGCAACTCGGTCGCCGCATACACTATTCTCAGAATGACTTGGTTGAGTACTCACCAGTCACAGAAAAGCATCTTACGGATGGCATGACAGTAAGAGAATTATGTAGCGCTGCCATAACCATGAGTGATAACACTGCGGCCAACTTACTTCTGACAACGATCGGAGGACCGAAGGAGCTAACCGCTTTTTTGCACAACATGGGGGATCATGTAACTCGCCTTGATCGTTGGGAACCGGAGCTGAATGAAGCCATACCAAACGACGAGCGTGACACCACGATGCCTGTAGCAATGGCAACAACGTTGCGCAAACTATTAACTGGCGAACTACTTACTCTAGCTTCCCGGCAACAATTAATAGACTGGATGGAGGCGGATAAAGTTGCAGGACCACTTCTGCGCTCGGCCCTTCCGGCTGGCTGGTTTATTGCTGATAAATCTGGAGCCGGTGAGCGTGGGTCTCGCGGTATCATTGCAGCACTGGGGCCAGATGGTAAGCCCTCCCGTATCGTAGTTATCTACACGACGGGGAGTCAGGCAACTATGGATGAACGAAATAGACAGATCGCTGAGATAGGTGCCTCACTGATTAAGCATTGGTAA

**pRST.11C.AS3.4**

aagcttgtcgacgagctgttgacaattaatcatcggctcgtataatgtgtggaattgtgagcggataacaatttcacacaaggagatctagaatgagcaacgacgaaactgtagagaaagtcacccaacaagtgtcggaactaaaaagcacagatgttaaagagcaagtagttacaccttgggatgtggaaggtggggttgatgaacaaggtagagcccaaaatattgattacgacaaattgatcaaacaattcggtactaagccggtcaacgaagaaaccctgaagagattcaagcaagtgacgggtcgtgaaccacatcattttttgcgtaagggattgtttttcagtgagcgtgacttcactaaaatattagacctttacgaacaaggcaaaccatttttcctatacactggtagaggtccttcgagcgattctatgcacttgggtcatatgatcccttttgtcttcaccaaatggttacaggaagtgtttgacgtaccattagtcatagagttgacagatgacgaaaaatttttattcaaacacaagttgaccatcaatgacgttaagaattttgcccgtgaaaatgccaaggatatcattgctgttggctttgatccaaagaacacctttatcttttctgatttgcaatacatgggtggtgcattttacgaaactgtagtaagagtttccagacaaattacaggatccactgcaaaggctgttttcgggtttaatgactccgactgtattggtaagttccattttgcctccattcaaattgctaccgcattcccaagctcatttcctaatgtgttaggcttgcctgataagacaccatgtttgattccatgtgcaattgaccaagatccatatttcagagtttgtagggatgtcgcggataaattgaagtactccaaacctgctttgcttcattccagattctttccagctttgcaaggttccacgaccaaaatgtcagcctctgatgataccactgccattttcatgaccgatacaccaaagcaaattcaaaagaaaattaacaagtacgcattcagcggtggtcaagtgtccgccgacctacatagagaattaggtggtaatcccgatgtcgatgttgcataccaatacttgtcatttttcaaggatgacgatgttttcttgaaggaatgctatgacaaatataagtccggtgaattactatcaggtgaaatgaagaaactgtgtatcgagactctgcaagaattcgttaaggcgttccaggaacgcagagctcaggtggacgaagagacGttggacaaattcatggtcccacataagttggtttggggcgaaaaggaaagacttgtcgcacctaagccaaaaactaagcaagaaaagaagtaactcgagagagaatataaaaagccagattattaatccggcttttttattatttcggccgacgcgctgggctacgtcttgctggcgttcgcgacgcgaggctggatggccttccccattatgattcttctcgcttccggcggcatcgggatgcccgcgttgcaggccatgctgtccaggcaggtagatgacgaccatcagggacagcttcaaggatcgctcgcggctcttaccagcctaacttcgatcactggaccgctgatcgtcacggcgatttatgccgcctcggcgagcacatggaacgggttggcatggattgtaggcgccgccctataccttgtctgcctccccgcgttgcgtcgcggtgcatggagccgggccacctcgacctgaatggaagccggcggcacctcgctaacggattcaccactccaagaattggagccaatcaattcttgcggagaactgtgaatgcgcaaaccaacccttggcagaacatatccatcgcgtccgccatctccagcagccgcacgcggcgcatctcgggcagcgttgggtcctggccaatgcataccaaacctgcacgctagtttcctgatgaacatttttccagcaattacacctctgtcgataattaactattgacgaaaagctgaaaaccactagaatgcgcctccgtggtaccaattctttttaagaattgatggtatgaagcggtggctcaatggtagagctggcgcctctaaagcgccgggttgcaggttcaattcctgtccgtttcaccaattttgaaccccgcttcggcggggttttttgttttctgtacatttcgtcaccctcccttcgcaataaacgcccgtaatatggccacgggtgcgcatgatcgtgctcctgtcgttgaggacccggctaggctggcggggttgccttactggttagcagaatgaatcaccgatacgcgagcgaacgtgaagcgactgctgctgcaaaacgtctgcgacctgagcaacaacatgaatggtcttcggtttccgtgtttcgtaaagtctggaaacgcggaagtcagcgccctgcaccattatgttccggatctgcatcgcaggatgctgctggctaccctgtggaacacctacatctgtattaacgaagcgctggcattgaccctgagtgatttttctctggtcccgccgcatccataccgccagttgtttaccctcacaacgttccagtaaccgggcatgttcatcatcagtaacccgtatcgtgagcatcctctctcgtttcatcggtatcattacccccatgaacagaaatcccccttacacggaggcatcagtgaccaaacaggaaaaaaccgcccttaacatggcccgctttatcagaagccagacattaacgcttctggagaaactcaacgagctggacgcggatgaacaggcagacatctgtgaatcgcttcacgaccacgctgatgagctttaccgcagctgcctcgcgcgtttcggtgatgacggtgaaaacctctgacacatgcagctcccggagacggtcacagcttgtctgtaagcggatgccgggagcagacaagcccgtcagggcgcgtcagcgggtgttggcgggtgtcggggcgcagccatgacccagtcacgtagcgatagcggagtgtatactggcttaactatgcggcatcagagcagattgtactgagagtgcaccatatgcggtgtgaaataccgcacagatgcgtaaggagaaaataccgcatcaggcgctcttccgcttcctcgctcactgactcgctgcgctcggtcgttcggctgcggcgagcggtatcagctcactcaaaggcggtaatacggttatccacagaatcaggggataacgcaggaaagaacatgtgagcaaaaggccagcaaaaggccaggaaccgtaaaaaggccgcgttgctggcgtttttccataggctccgcccccctgacgagcatcacaaaaatcgacgctcaagtcagaggtggcgaaacccgacaggactataaagataccaggcgtttccccctggaagctccctcgtgcgctctcctgttccgaccctgccgcttaccggatacctgtccgcctttctcccttcgggaagcgtggcgctttctcatagctcacgctgtaggtatctcagttcggtgtaggtcgttcgctccaagctgggctgtgtgcacgaaccccccgttcagcccgaccgctgcgccttatccggtaactatcgtcttgagtccaacccggtaagacacgacttatcgccactggcagcagccactggtaacaggattagcagagcgaggtatgtaggcggtgctacagagttcttgaagtggtggcctaactacggctacactagaaggacagtatttggtatctgcgctctgctgaagccagttaccttcggaaaaagagttggtagctcttgatccggcaaacaaaccaccgctggtagcggtggtttttttgtttgcaagcagcagattacgcgcagaaaaaaaggatctcaagaagatcctttgatcttttctacggggtctgacgctcagtggaacgaaaactcacgttaagggattttggtcatgagattatcaaaaaggatcttcacctagatccttttaaattaaaaatgaagttttaaatcaatctaaagtatatatgagtaaacttggtctgacagttaccaatgcttaatcagtgaggcacctatctcagcgatctgtctatttcgttcatccatagttgcctgactccccgtcgtgtagataactacgatacgggagggcttaccatctggccccagtgctgcaatgataccgcgGgacccacgctcaccggctccagatttatcagcaataaaccagccagccggaagggccgagcgcagaagtggtcctgcaactttatccgcctccatccagtctattaattgttgccgggaagctagagtaagtagttcgccagttaatagtttgcgcaacgttgttgccattgctgcaggcatcgtggtgtcacgctcgtcgtttggtatggcttcattcagctccggttcccaacgatcaaggcgagttacatgatcccccatgttgtgcaaaaaagcggttagctccttcggtcctccgatcgttgtcagaagtaagttggccgcagtgttatcactcatggttatggcagcactgcataattctcttactgtcatgccatccgtaagatgcttttctgtgactggtgagtactcaaccaagtcattctgagaatagtgtatgcggcgaccgagttgctcttgcccggcgtcaacacgggataataccgcgccacatagcagaactttaaaagtgctcatcattggaaaacgttcttcggggcgaaaactctcaaggatcttaccgctgttgagatccagttcgatgtaacccactcgtgcacccaactgatcttcagcatcttttactttcaccagcgtttctgggtgagcaaaaacaggaaggcaaaatgccgcaaaaaagggaataagggcgacacggaaatgttgaatactcatactcttcctttttcaatattattgaagcatttatcagggttattgtctcatgagcggatacatatttgaatgtatttagaaaaataaacaaataggggttccgcgcacatttccccgaaaagtgccacctgacgtcccgacaccatcgaatggtgcaaaacctttcgcggtatggcatgatagcgcccggaagagagtcaattcagggtggtgaatgtgaaaccagtaacgttatacgatgtcgcagagtatgccggtgtctcttatcagaccgtttcccgcgtggtgaaccaggccagccacgtttctgcgaaaacgcgggaaaaagtggaagcggcgatggcggagctgaattacattcccaaccgcgtggcacaacaactggcgggcaaacagtcgttgctgattggcgttgccacctccagtctggccctgcacgcgccgtcgcaaattgtcgcggcgattaaatctcgcgccgatcaactgggtgccagcgtggtggtgtcgatggtagaacgaagcggcgtcgaagcctgtaaagcggcggtgcacaatcttctcgcgcaacgcgtcagtgggctgatcattaactatccgctggatgaccaggatgccattgctgtggaagctgcctgcactaatgttccggcgttatttcttgatgtctctgaccagacacccatcaacagtattattttctcccatgaagacggtacgcgactgggcgtggagcatctggtcgcattgggtcaccagcaaatcgcgctgttagcgggcccattaagttctgtctcggcgcgtctgcgtctggctggctggcataaatatctcactcgcaatcaaattcagccgatagcggaacgggaaggcgactggagtgccatgtccggttttcaacaaaccatgcaaatgctgaatgagggcatcgttcccactgcgatgctggttgccaacgatcagatggcgctgggcgcaatgcgcgccattaccgagtccgggctgcgcgttggtgcggatatctcggtagtgggatacgacgataccgaagacagctcatgttatatcccgccgttaaccaccatcaaacaggattttcgcctgctggggcaaaccagcgtggaccgcttgctgcaactctctcagggccaggcggtgaagggcaatcagctgttgcccgtctcactggtgaaaagaaaaaccaccctggcgcccaatacgcaaaccgcctctccccgcgcgttggccgattcattaatgcagctggcacgacaggtttcccgactggaaagcgggcagtgagcgcaacgcaattaatgtaagttagctcactcattaggcacaattctcatgtttgacagcttatcatcgactgcacggtgcaccaatgcttctggcgtcaggcagccatcggaagctgtggtatggctgtgcaggtcgtaaatcactgcataattcgtgtcgctcaaggcgca

**pBAD30b2_T7RSS**

AACTAAGTTACCAAACGCAACGTAGTTTTATAAAATGGGCTCCAGCCACCACCACCACCACCACGGATCTAACACCATAAACATAGCAAAGAATGACTTTAGTGATATAGAACTGGCGGCTATACCATTCAACACCCTGGCGGACCACTATGGAGAACGCCTCGCTCGGGAGCAACTGGCCCTGGAACACGAAAGCTATGAGATGGGAGAGGCTCGGTTTCGCAAAATGTTCGAAAGACAGCTCAAAGCTGGAGAAGTGGCTGATAATGCAGCAGCAAAGCCACTGATAACAACCCTTCTCCCAAAGATGATAGCAAGGATAAACGATTGGTTCGAAGAAGTGAAAGCAAAAAGAGGCAAACGGCCTACAGCTTTTCAATTCCTCCAAGAGATAAAGCCTGAAGCAGTGGCCTACATAACAATCAAAACAACCCTTGCTTGCCTGACAAGCGCAGATAACACAACGGTGCAAGCAGTGGCATCAGCAATAGGGAGAGCGATCGAAGAtGAAGCACGGTTTGGCAGAATCAGAGAtCTCGAAGCAAAACACTTTAAGAAGAATGTGGAAGAACAACTTAACAAAAGAGTGGGACATGTGTACAAAAAGGCATTCATGCAAGTGGTGGAAGCGGATATGCTGAGTAAAGGTCTGCTAGGTGGAGAAGCGTGGAGTAGTTGGCATAAAGAGGACAGTATACACGTGGGTGTGCGCTGCATAGAGATGTTGATCGAAAGTACGGGAATGGTCAGCTTGCATAGGCAGAATGCAGGAGTGGTGGGACAGGACAGCGAGACTATAGAACTCGCACCAGAATACGCAGAAGCCATCGCAACAAGAGCGGGAGCTCTAGCTGGGATCAGCCCCATGTTCCAACCCTGTGTGGTTCCACCAAAACCATGGACAGGGATAACTGGAGGAGGATACTGGGCAAATGGGAGACGACCACTAGCCCTGGTGAGGACACATAGCAAGAAAGCGCTCATGAGATACGAAGATGTGTACATGCCAGAAGTGTACAAGGCAATCAACATAGCACAGAACACGGCCTGGAAAATCAATAAGAAAGTGTTGGCCGTGGCGAATGTCATCACTAAATGGAAGCACTGTCCCGTGGAGGATATACCAGCAATAGAAAGAGAGGAATTACCTATGAAACCAGAGGATATAGATATGAATCCGGAAGCGCTGACGGCGTGGAAGAGGGCAGCGGCAGCGGTGTACAGAAAGGATAAAGCGCGAAAATCACGAAGAATTAGCCTGGAGTTTATGCTGGAACAAGCGAATAAGTTCGCAAATCACAAAGCAATATGGTTTCCATACAATATGGATTGGAGAGGGAGGGTGTATGCAGTGAGTATGTTTAACCCACAAGGAAATGATATGACAAAAGGACTATTGACCCTGGCGAAAGGAAAACCGATAGGAAAAGAAGGATACTACTGGCTTAAGATACATGGCGCAAACTGTGCAGGCGTGGATAAAGTGCCATTTCCCGAAAGGATAAAGTTCATAGAAGAGAACCACGAGAACATCATGGCATGCGCAAAAAGTCCACTGGAGAACACATGGTGGGCAGAACAAGACAGCCCGTTTTGCTTTTTGGCTTTCTGCTTTGAGTATGCGGGAGTGCAACACCACGGACTATCATACAACTGCTCACTCCCACTGGCTTTTGACGGTAGTTGTAGTGGAATACAACACTTCTCAGCCATGTTGAGGGATGAGGTGGGTGGAAGgGCAGTCAATCTCCTTCCCTCCGAAACAGTCCAAGATATCTACGGAATAGTGGCAAAGAAAGTGAACGAAATCCTTCAAGCGGACGCGATAAACGGAACAGACAATGAGGTGGTGACAGTGACAGACGAGAATACAGGAGAAATCAGTGAAAAAGTTAAACTGGGAACAAAAGCCCTAGCGGGACAATGGCTCGCATATGGCGTGACTAGGAGTGTGACAAAAAGAAGCGTGATGACACTAGCATACGGAAGCAAAGAATTTGGTTTTAGGCAACAAGTGCTGGAAGATACAATACAACCAGCGATAGACTCAGGAAAAGGACTAATGTTTACACAACCCAATCAAGCGGCAGGATACATGGCAAAACTGATATGGGAATCCGTGTCAGTGACAGTGGTGGCGGCAGTGGAAGCAATGAATTGGCTGAAGAGTGCGGCAAAGCTGCTGGCAGCGGAAGTGAAAGACAAAAAGACAGGAGAAATCCTACGGAAAAGGTGTGCGGTGCATTGGGTAACGCCTGATGGGTTCCCAGTGTGGCAAGAATACAAAAAGCCAATACAAACAAGACTTAACCTAATGTTCCTAGGACAGTTTAGACTACAACCAACCATAAACACAAACAAAGATTCAGAAATAGACGCACACAAACAAGAAAGTGGGATAGCACCAAACTTCGTGCACTCACAAGACGGAAGCCACCTCCGCAAAACAGTGGTCTGGGCACATGAAAAGTATGGTATTGAATCATTCGCGCTGATACATGATAGCTTCGGAACGATACCCGCAGACGCAGCCAACTTGTTTAAGGCAGTGCGAGAAACGATGGTGGATACATATGAGTCATGCGACGTGCTTGCCGACTTTTACGATCAGTTCGCGGATCAACTACATGAAAGCCAACTCGACAAAATGCCAGCACTACCCGCAAAAGGAAACCTGAACTTGCGCGATATACTTGAAAGCGATTTTGCATTTGCCTGATAAAGCTCGAGATCTGCAGCTGGTACCATATGGGAATTCGAAGCTTGGCTGTTTTGGCGGATGAGAGAAGATTTTCAGCCTGATACAGATTAAATCAGAACGCAGAAGCGGTCTGATAAAACAGAATTTGCCTGGCGGCAGTAGCGCGGTGGTCCCACCTGACCCCATGCCGAACTCAGAAGTGAAACGCCGTAGCGCCGATGGTAGTGTGGCTCTTCCCCATGCCGAACTCAGAAGTGAAACGCCGTAGCGCCGATGGTAGTGTGGCTCTTCCCCATGCCGAACTCAGAAGTGAAACGCCGTAGCGCCGATGGTAGTGTGGCTAGTCCCCATGCGAGAGTAGGGAACTGCCAGGCATCAAATAAAACGAAAGGCTCAGTCGAAAGACTGGGCCTTTCGTTTTATCTGTTGTTTGTCGGTGAACGCTCTCCTGAGTAGGACAAATCCGCCGGGAGCGGATTTGAACGTTGCGAAGCAACGGCCCGGAGGGTGGCGGGCAGGACGCCCGCCATAAACTGCCAGGCATCAAATTAAGCAGAAGGCCATCCTGACGGATGGCCTTTTTGCGTTTCTACAAACTCTTTTGTTTATTTTTCTAAATACATTCAAATATGTATCCGCTCATGAGACAATAACCCTGATAAATGCTTCAATAATATTGAAAAAGGAAGAGTATGAGTATTCAACATTTCCGTGTCGCCCTTATTCCCTTTTTTGCGGCATTTTGCCTTCCTGTTTTTGCTCACCCAGAAACGCTGGTGAAAGTAAAAGATGCTGAAGATCAGTTGGGTGCACGAGTGGGTTACATCGAACTGGATCTCAACAGCGGTAAGATCCTTGAGAGTTTTCGCCCCGAAGAACGTTTTCCAATGATGAGCACTTTTAAAGTTCTGCTATGTGGCGCGGTATTATCCCGTGTTGACGCCGGGCAAGAGCAACTCGGTCGCCGCATACACTATTCTCAGAATGACTTGGTTGAGTACTCACCAGTCACAGAAAAGCATCTTACGGATGGCATGACAGTAAGAGAATTATGCAGTGCTGCCATAACCATGAGTGATAACACTGCGGCCAACTTACTTCTGACAACGATCGGAGGACCGAAGGAGCTAACCGCTTTTTTGCACAACATGGGGGATCATGTAACTCGCCTTGATCGTTGGGAACCGGAGCTGAATGAAGCCATACCAAACGACGAGCGTGACACCACGATGCCTGCAGCAATGGCAACAACGTTGCGCAAACTATTAACTGGCGAACTACTTACTCTAGCTTCCCGGCAACAATTAATAGACTGGATGGAGGCGGATAAAGTTGCAGGACCACTTCTGCGCTCGGCCCTTCCGGCTGGCTGGTTTATTGCTGATAAATCTGGAGCCGGTGAGCGTGGGagcCGCGGTATCATTGCAGCACTGGGGCCAGATGGTAAGCCCTCCCGTATCGTAGTTATCTACACGACGGGGAGTCAGGCAACTATGGATGAACGAAATAGACAGATCGCTGAGATAGGTGCCTCACTGATTAAGCATTGGTAACTGTCAGACCAAGTTTACTCATATATACTTTAGATTGATTTACGCGCCCTGTAGCGGCGCATTAAGCGCGGCGGGTGTGGTGGTTACGCGCAGCGTGACCGCTACACTTGCCAGCGCCCTAGCGCCCGCTCCTTTCGCTTTCTTCCCTTCCTTTCTCGCCACGTTCGCCGGCTTTCCCCGTCAAGCTCTAAATCGGGGGCTCCCTTTAGGGTTCCGATTTAGTGCTTTACGGCACCTCGACCCCAAAAAACTTGATTTGGGTGATGGTTCACGTAGTGGGCCATCGCCCTGATAGACGGTTTTTCGCCCTTTGACGTTGGAGTCCACGTTCTTTAATAGTGGACTCTTGTTCCAAACTTGAACAACACTCAACCCTATCTCGGGCTATTCTTTTGATTTATAAGGGATTTTGCCGATTTCGGCCTATTGGTTAAAAAATGAGCTGATTTAACAAAAATTTAACGCGAATTTTAACAAAATATTAACGTTTACAATTTAAAAGGATCTAGGTGAAGATCCTTTTTGATAATCTCATGACCAAAATCCCTTAACGTGAGTTTTCGTTCCACTGAGCGTCAGACCCCGTAGAAAAGATCAAAGGATCTTCTTGAGATCCTTTTTTTCTGCGCGTAATCTGCTGCTTGCAAACAAAAAAACCACCGCTACCAGCGGTGGTTTGTTTGCCGGATCAAGAGCTACCAACTCTTTTTCCGAAGGTAACTGGCTTCAGCAGAGCGCAGATACCAAATACTGTCCTTCTAGTGTAGCCGTAGTTAGGCCACCACTTCAAGAACTCTGTAGCACCGCCTACATACCTCGCTCTGCTAATCCTGTTACCAGTCAGGCATTTGAGAAGCACACGGTCACACTGCTTCCGGTAGTCAATAAACCGGTAAACCAGCAATAGACATAAGCGGCTATTTAACGACCCTGCCCTGAACCGACGACCGGGTCGAATTTGCTTTCGAATTTCTGCCATTCATCCGCTTATTATCACTTATTCAGGCGTAGCACCAGGCGTTTAAGGGCACCAATAACTGCCTTAAAAAAATTACGCCCCGCCCTGCCACTCATCGCAGTACTGTTGTAATTCATTAAGCATTCTGCCGACATGGAAGCCATCACAGACGGCATGATGAACCTGAATCGCCAGCGGCATCAGCACCTTGTCGCCTTGCGTATAATATTTGCCGCTAGCGGAGTGTATACTGGCTTACTATGTTGGCACTGATGAGGGTGTCAGTGAAGTGCTTCATGTGGCAGGAGAAAAAAGGCTGCACCGGTGCGTCAGCAGAATATGTGATACAGGATATATTCCGCTTCCTCGCTCACTGACTCGCTACGCTCGGTCGTTCGACTGCGGCGAGCGGAAATGGCTTACGAACGGGGCGGAGATTTCCTGGAAGATGCCAGGAAGATACTTAACAGGGAAGTGAGAGGGCCGCGGCAAAGCCGTTTTTCCATAGGCTCCGCCCCCCTGACAAGCATCACGAAATCTGACGCTCAAATCAGTGGTGGCGAAACCCGACAGGACTATAAAGATACCAGGCGTTTCCCCCTGGCGGCTCCCTCGTGCGCTCTCCTGTTCCTGCCTTTCGGTTTACCGGTGTCATTCCGCTGTTATGGCCGCGTTTGTCTCATTCCACGCCTGACACTCAGTTCCGGGTAGGCAGTTCGCTCCAAGCTGGACTGTATGCACGAACCCCCCGTTCAGTCCGACCGCTGCGCCTTATCCGGTAACTATCGTCTTGAGTCCAACCCGGAAAGACATGCAAAAGCACCACTGGCAGCAGCCACTGGTAATTGATTTAGAGGAGTTAGTCTTGAAGTCATGCGCCGGTTAAGGCTAAACTGAAAGGACAAGTTTTGGTGACTGCGCTCCTCCAAGCCAGTTACCTCGGTTCAAAGAGTTGGTAGCTCAGAGAACCTTCGAAAAACCGCCCTGCAAGGCGGTTTTTTCGTTTTCAGAGCAAGAGATTACGCGCAGACCAAAACGATCTCAAGAAGATCATCTTATTAATCAGATAAAATATTTGCTCATGAGCCCGAAGTGGCGAGCCCGATCTTCCCCATCGGTGATGTCGGCGATATAGGCGCCAGCAACCGCACCTGTGGCGCCGGTGATGCCGGCCACGATGCGTCCGGCGTAGAGGATCTGCTCATGTTTGACAGCTTATCATCGATGCATAATGTGCCTGTCAAATGGACGAAGCAGGGATTCTGCAAACCCTATGCTACTCCGTCAAGCCGTCAATTGTCTGATTCGTTACCAATTATGACAACTTGACGGCTACATCATTCACTTTTTCTTCACAACCGGCACGGAACTCGCTCGGGCTGGCCCCGGTGCATTTTTTAAATACCCGCGAGAAATAGAGTTGATCGTCAAAACCAACATTGCGACCGACGGTGGCGATAGGCATCCGGGTGGTGCTCAAAAGCAGCTTCGCCTGGCTGATACGTTGGTCCTCGCGCCAGCTTAAGACGCTAATCCCTAACTGCTGGCGGAAAAGATGTGACAGACGCGACGGCGACAAGCAAACATGCTGTGCGACGCTGGCGATATCAAAATTGCTGTCTGCCAGGTGATCGCTGATGTACTGACAAGCCTCGCGTACCCGATTATCCATCGGTGGATGGAGCGACTCGTTAATCGCTTCCATGCGCCGCAGTAACAATTGCTCAAGCAGATTTATCGCCAGCAGCTCCGAATAGCGCCCTTCCCCTTGCCCGGCGTTAATGATTTGCCCAAACAGGTCGCTGAAATGCGGCTGGTGCGCTTCATCCGGGCGAAAGAACCCCGTATTGGCAAATATTGACGGCCAGTTAAGCCATTCATGCCAGTAGGCGCGCGGACGAAAGTAAACCCACTGGTGATACCATTCGCGAGCCTCCGGATGACGACCGTAGTGATGAATCTCTCCTGGCGGGAACAGCAAAATATCACCCGGTCGGCAAACAAATTCTCGTCCCTGATTTTTCACCACCCCCTGACCGCGAATGGTGAGATTGAGAATATAACCTTTCATTCCCAGCGGTCGGTCGATAAAAAAATCGAGATAACCGTTGGCCTCAATCGGCGTTAAACCCGCCACCAGATGGGCATTAAACGAGTATCCCGGCAGCAGGGGATCATTTTGCGCTTCAGCCATACTTTTCATACTCCCGCCATTCAGAGAAGAAACCAATTGTCCATATTGCATCAGACATTGCCGTCACTGCGTCTTTTACTGGCTCTTCTCGCTAACCAAACCGGTAACCCCGCTTATTAAAAGCATTCTGTAACAAAGCGGGACCAAAGCCATGACAAAAACGCGTAACAAAAGTGTCTATAATCACGGCAGAAAAGTCCACATTGATTATTTGCACGGCGTCACACTTTGCTATGCCATAGCATTTTTATCCATAAGATTAGCGGATCCTACCTGACGCTTTTTATCGCAACTCTCTACTGTTTCTCCATACCCGTTTTTTGGGCTAACAGGAGGAATTAACC

**pET29aΔΔΔ_TgoT**

atgattctggacaccgactacataaccgaagacggcaaaccggtgatccgcatcttcaaaaaggagaacggcgagtttaagatcgattacgaccgcaacttcgagccgtacatttacgccctgctgaaggacgatagtgccatcgaggacgtgaagaagattaccgcagaacgtcacggcacaacagtgcgtgtggtgcgtgccgagaaggtgaagaaaaaattcctgggccgcccgatcgaggtgtggaaactgtactttacccacccgcaagaccagccggccattcgcgacaaaattaaggaacacccggccgttgtggacatttacgagtacgacattccgttcgcaaagcgttacctgatcgacaagggcctgatcccgatggaaggtgacgaagagctgaagatgctggcctttgccatcgccacgctgtaccatgagggcgaggaattcgccgaaggcccgatcctgatgatcagctatgcagatgaagagggcgcacgtgtgatcacatggaaaaatatcgatctgccgtacgttgatgtggttagcaccgaaaaagaaatgatcaagcgttttctgaaggttgttaaggagaaagacccggacgtgctgatcacctacaacggtgacaactttgattttgcctatctgaagaagcgtagcgagaaactgggcgttaaattcattctgggtcgcgagggtagcgagcctaagatccagcgtatgggtgatcgcttcgccgttgaagtgaaaggccgcatccacttcgatctgtatcctgtgatccgccgcaccatcaacctgccgacctacaccctggaggcagtttacgaagccattttcggtcagccgaaggaaaaggtttatgccgaagagatcgcccaggcatgggaaaccggtgaaggcctggagcgtgtggcccgttacagcatggaagacgccaaggtgacctatgaactgggcaaagagttttttccgatggaggcacaactgagccgcctggttggccagagcctgtgggatgtgagccgcagtagcacaggtaatctggttgaatggttcctgctgcgtaaagcatacgaacgcaatgaactggccccgaacaaacctgacgaacgtgaattagcccgccgtcgcgagagttatgccggtggttacgtgaaagaaccggaacgtggcctgtgggagaacattgtgtatctggactttcgtagcttataccctagcattatcattacccacaacgtgagcccggatacactgaatcgtgagggctgcgaggaatacgatgttgccccgcaggtgggccataagttctgcaaggatttcccgggctttatcccgagcctgctgggcgatctgctggaagagcgtcaaaaggttaaaaagaaaatgaaagccacaatcgacccgatcgaaaagaagctgctggattaccgccagcgcttaatcaagattctggcaaatagtttttatggttattacggctatgccaaagcacgctggtattgtaaggagtgcgcagaaagcgttacagcctggggtcgtcagtatatcgagacgacaattcgcgaaatcgaagaaaaatttggcttcaaagttctgtatgccgataccgacggcttctttgccaccattcctggtgccgacgcagagacagttaaaaagaaggccaaagaatttttagattacattaacgccaaattaccgggcctgctggaactggaatatgagggcttctacaaacgcgggttcttcgttaccaagaagaagtacgcagtgatcgacgaggaagacaagattaccacccgtggcctggaaatcgttcgccgcgattggagcgaaattgcaaaggagacacaggcacgcgtgctggaggccatcttaaaacatggcgacgttgaagaggcggttcgtatcgtgaaagaggtgacagagaagctgagtaagtatgaagtgccgccggagaagctggttatctatgagcagatcacccgcgatctgaaggactataaagccacaggcccgcatgttgcagttgccaagcgtctggccgcacgcggcattaagatccgtccgggcaccgttatcagctacatcgtgctgaaaggcagtggtcgcattGgcgaccgcgcaattccgttcgatgaatttgatccggccaaacacaaatatgacgccgagtattacattgaaaatcaggttctgccggcagtggaacgcattttacgcgcattcggctatcgcaaagaggacctgcgctatcagaagacacgtcaggttggtctgggtgcatggctgaagccgaaaaccGGTGGCTCAGGCGGTCACCACCACCACCACCACTGAGATCCGGCTGCTAACAAAGCCCGAAAGGAAGCTGAGTTGGCTGCTGCCACCGCTGAGCAATAACTAGCATAACCCCTTGGGGCCTCTAAACGGGTCTTGAGGGGTTTTTTGCTGAAAGctGAGGAACTATATCCGGATTGGCGAATGGGACGCGCCCTGTAGCGGCGCATTAAGCGCGGCGGGTGTGGTGGTTACGCGCAGCGTGACCGCTACACTTGCCAGCGCCCTAGCGCCCGCTCCTTTCGCTTTCTTCCCTTCCTTTCTCGCCACGTTCGCCGGCTTTCCCCGTCAAGCTCTAAATCGGGGGCTCCCTTTAGGGTTCCGATTTAGTGCTTTACGGCACCTCGACCCCAAAAAACTTGATTAGGGTGATGGTTCACGTAGTGGGCCATCGCCCTGATAGACGGTTTTTCGCCCTTTGACGTTGGAGTCCACGTTCTTTAATAGTGGACTCTTGTTCCAAACTGGAACAACACTCAACCCTATCTCGGTCTATTCTTTTGATTTATAAGGGATTTTGCCGATTTCGGCCTATTGGTTAAAAAATGAGCTGATTTAACAAAAATTTAACGCGAATTTTAACAAAATATTAACGTTTACAATTTCAGGTGGCACTTTTCGGGGAAATGTGCGCGGAACCCCTATTTGTTTATTTTTCTAAATACATTCAAATATGTATCCGCTCATGAATTAATTCTTAGAAAAACTCATCGAGCATCAAATGAAACTGCAATTTATTCATATCAGGATTATCAATACCATATTTTTGAAAAAGCCGTTTCTGTAATGAAGGAGAAAACTCACCGAGGCAGTTCCATAGGATGGCAAGATCCTGGTATCGGTCTGCGATTCCGACTCGTCCAACATCAATACAACCTATTAATTTCCCCTCGTCAAAAATAAGGTTATCAAGTGAGAAATCACCATGAGTGACGACTGAATCCGGTGAGAATGGCAAAAGTTTATGCATTTCTTTCCAGACTTGTTCAACAGGCCAGCCATTACGCTCGTCATCAAAATCACTCGCATCAACCAAACCGTTATTCATTCGTGATTGCGCCTGAGCGAGACGAAATACGCGATCGCTGTTAAAAGGACAATTACAAACAGGAATCGAATGCAACCGGCGCAGGAACACTGCCAGCGCATCAACAATATTTTCACCTGAATCAGGATATTCTTCTAATACCTGGAATGCTGTTTTCCCGGGGATCGCAGTGGTGAGTAACCATGCATCATCAGGAGTACGGATAAAATGCTTGATGGTCGGAAGAGGCATAAATTCCGTCAGCCAGTTTAGTCTGACCATCTCATCTGTAACATCATTGGCAACGCTACCTTTGCCATGTTTCAGAAACAACTCTGGCGCATCGGGCTTCCCATACAATCGATAGATTGTCGCACCTGATTGCCCGACATTATCGCGAGCCCATTTATACCCATATAAATCAGCATCCATGTTGGAATTTAATCGCGGCCTAGAGCAAGACGTTTCCCGTTGAATATGGCTCATAACACCCCTTGTATTACTGTTTATGTAAGCAGACAGTTTTATTGTTCATGACCAAAATCCCTTAACGTGAGTTTTCGTTCCACTGAGCGTCAGACCCCGTAGAAAAGATCAAAGGATCTTCTTGAGATCCTTTTTTTCTGCGCGTAATCTGCTGCTTGCAAACAAAAAAACCACCGCTACCAGCGGTGGTTTGTTTGCCGGATCAAGAGCTACCAACTCTTTTTCCGAAGGTAACTGGCTTCAGCAGAGCGCAGATACCAAATACTGTCCTTCTAGTGTAGCCGTAGTTAGGCCACCACTTCAAGAACTCTGTAGCACCGCCTACATACCTCGCTCTGCTAATCCTGTTACCAGTGGCTGCTGCCAGTGGCGATAAGTCGTGTCTTACCGGGTTGGACTCAAGACGATAGTTACCGGATAAGGCGCAGCGGTCGGGCTGAACGGGGGGTTCGTGCACACAGCCCAGCTTGGAGCGAACGACCTACACCGAACTGAGATACCTACAGCGTGAGCTATGAGAAAGCGCCACGCTTCCCGAAGGGAGAAAGGCGGACAGGTATCCGGTAAGCGGCAGGGTCGGAACAGGAGAGCGCACGAGGGAGCTTCCAGGGGGAAACGCCTGGTATCTTTATAGTCCTGTCGGGTTTCGCCACCTCTGACTTGAGCGTCGATTTTTGTGATGCTCGTCAGGGGGGCGGAGCCTATGGAAAAACGCCAGCAACGCGGCCTTTTTACGGTTCCTGGCCTTTTGCTGGCCTTTTGCTCACATGTTCTTTCCTGCGTTATCCCCTGATTCTGTGGATAACCGTATTACCGCCTTTGAGTGAGCTGATACCGCTCGCCGCAGCCGAACGACCGAGCGCAGCGAGTCAGTGAGCGAGGAAGCTCCTtgatgcctccgtgtaagggGGATTTCTGTTCATGGGGGTAATGATACCGATGAAACGAGAGAGGATGCTCACGATACGGGTTACTGATGATGAACATGCCCGGTTACTGGAACGTTGTGAGGGTAAACAACTGGCGGTATGGATGCGGCGGGACCAGAGAAAAATCACTCAGGGTCAATGCCAGCGCTTCGTTAATACAGATGTAGGTGTTCCACAGGGTAGCCAGCAGCATCCTGCGATGCAGATCCGGAACATAATGGTGCAGGGCGCTGACTTCCGCGTTTCCAGACTTTACGAAACACGGAAACCGAAGACCATTCATGTTGTTGCTCAGGTCGCAGACGTTTTGCAGCAGCAGTCGCTTCACGTTCGCTCGCGTATCGGTGATTCATTCTGCTAACCAGTAAGGCAACCCCGCCAGCCTAGCCGGGTCCTCAACGACAGGAGCACGATCATGCGCACCCGTGGGGCCGCCATGCCGGCGATAATGGCCTGCTTCTCGCCGAAACGTTTGGTGGCGGGACCAGTGACGAAGGCTTGAGCGAGGGCGTGCAAGATTCCGAATACCGCAAGCGACAGGCCGATCATCGTCGCGCTCCAGCGAAAGCGGTCCTCGCCGAAAATGACCCAGAGCGCTGCCGGCACCTGTCCTACGAGTTGCATGATAAAGAAGACAGTCATAAGTGCGGCGACGATAGTCATGCCCCGCGCCCACCGGAAGGAGCTGACTGGGTTGAAGGCTCTCAAGGGCATCGGTCGAGATCCCGGTGCCTAATGAGTGAGCTAACTTACATTAATTGCGTTGCGCTCACTGCCCGCTTTCCAGTCGGGAAACCTGTCGTGCCAGCTGCATTAATGAATCGGCCAACGCGCGGGGAGAGGCGGTTTGCGTATTGGGCGCCAGGGTGGTTTTTCTTTTCACCAGTGAGACGGGCAACAGCTGATTGCCCTTCACCGCCTGGCCCTGAGAGAGTTGCAGCAAGCGGTCCACGCTGGTTTGCCCCAGCAGGCGAAAATCCTGTTTGATGGTGGTTAACGGCGGGATATAACATGAGCTGTCTTCGGTATCGTCGTATCCCACTACCGAGATGTCCGCACCAACGCGCAGCCCGGACTCGGTAATGGCGCGCATTGCGCCCAGCGCCATCTGATCGTTGGCAACCAGCATCGCAGTGGGAACGATGCCCTCATTCAGCATTTGCATGGTTTGTTGAAAACCGGACATGGCACTCCAGTCGCCTTCCCGTTCCGCTATCGGCTGAATTTGATTGCGAGTGAGATATTTATGCCAGCCAGCCAGACGCAGACGCGCCGAGACAGAACTTAATGGGCCCGCTAACAGCGCGATTTGCTGGTGACCCAATGCGACCAGATGCTCCACGCCCAGTCGCGTACCGTCTTCATGGGAGAAAATAATACTGTTGATGGGTGTCTGGTCAGAGACATCAAGAAATAACGCCGGAACATTAGTGCAGGCAGCTTCCACAGCAATGGCATCCTGGTCATCCAGCGGATAGTTAATGATCAGCCCACTGACGCGTTGCGCGAGAAGATTGTGCACCGCCGCTTTACAGGCTTCGACGCCGCTTCGTTCTACCATCGACACCACCACGCTGGCACCCAGTTGATCGGCGCGAGATTTAATCGCCGCGACAATTTGCGACGGCGCGTGCAGGGCCAGACTGGAGGTGGCAACGCCAATCAGCAACGACTGTTTGCCCGCCAGTTGTTGTGCCACGCGGTTGGGAATGTAATTCAGCTCCGCCATCGCCGCTTCCACTTTTTCCCGCGTTTTCGCAGAAACGTGGCTGGCCTGGTTCACCACGCGGGAAACGGTCTGATAAGAGACACCGGCATACTCTGCGACATCGTATAACGTTACTGGTTTCACATTCACCACCCTGAATTGACTCTCTTCCGGGCGCTATCATGCCATACCGCGAAAGGTTTTGCGCCATTCGATGGTGTCCGGGATCTCGACGCTCTCCCTTATGCGACTCCTGCATTAGGAAGCAGCCCAGTAGTAGGTTGAGGCCGTTGAGCACCGCCGCCGCAAGGAATGGTGCATGCAAGGAGATGGCGCCCAACAGTCCCCCGGCCACGGGGCCTGCCACCATACCCACGCCGAAACAAGCGCTCATGAGCCCGAAGTGGCGAGCCCGATCTTCCCCATCGGTGATGTCGGCGATATAGGCGCCAGCAACCGCACCTGTGGCGCCGGTGATGCCGGCCACGATGCGTCCGGCGTAGAGGATCGAGATCGATCTCGATCCCGCGAAATTAATACGACTCACTATAGGGGAATTGTGAGCGGATAACAATTCCCCTCTAGAAATAATTTTGTTTAACTTTAAGAAGGAGATATACACTTCA

**pGDR11b_KOD**

ATGATCCTCGACACTGACTACATAACCGAGGATGGAAAGCCTGTCATAAGAATTTTCAAGAAGGAAAACGGCGAGTTTAAGATTGAGTACGACCGGACTTTTGAACCCTACTTCTACGCCCTCCTGAAGGACGATTCTGCCATTGAGGAAGTCAAGAAGATAACCGCCGAGAGGCACGGGACGGTTGTAACGGTTAAGCGGGTTGAAAAGGTTCAGAAGAAGTTCCTCGGGAGACCAGTTGAGGTCTGGAAACTCTACTTTACTCATCCGCAGGACGTCCCAGCGATAAGGGACAAGATACGAGAGCATCCAGCAGTTATTGACATCTACGAGTACGACATACCCTTCGCCAAGCGCTACCTCATAGACAAGGGATTAGTGCCAATGGAAGGCGACGAGGAGCTGAAAATGCTCGCCTTCGCGATTGCGACTCTCTACCATGAGGGCGAGGAGTTCGCCGAGGGGCCAATCCTTATGATAAGCTACGCCGACGAGGAAGGGGCCAGGGTGATAACTTGGAAGAACGTGGATCTCCCCTACGTTGACGTCGTCTCGACGGAGAGGGAGATGATAAAGCGCTTCCTCCGTGTTGTGAAGGAGAAAGACCCGGACGTTCTCATAACCTACAACGGCGACAACTTCGACTTCGCCTATCTGAAAAAGCGCTGTGAAAAGCTCGGAATAAACTTCGCCCTCGGAAGGGATGGAAGCGAGCCGAAGATTCAGAGGATGGGCGACAGGTTTGCCGTCGAAGTGAAGGGACGGATACACTTCGATCTCTATCCTGTGATAAGACGGACGATAAACCTGCCCACATACACGCTTGAGGCCGTTTATGAAGCCGTCTTCGGTCAGCCGAAGGAGAAGGTTTACGCTGAGGAAATAACCACAGCCTGGGAAACCGGCGAGAACCTTGAGAGAGTCGCCCGCTACTCGATGGAAGATGCGAAGGTCACATACGAGCTTGGGAAGGAGTTCCTTCCGATGGAGGCCCAGCTTTCTCGCTTAATCGGCCAGTCCCTCTGGGACGTCTCCCGCTCCAGCACTGGCAACCTCGTTGAGTGGTTCCTCCTCAGGAAGGCCTATGAGAGGAATGAGCTGGCCCCGAACAAGCCCGATGAAAAGGAGCTGGCCAGAAGACGGCAGAGCTATGAAGGAGGCTATGTAAAAGAGCCCGAGAGAGGGTTGTGGGAGAACATAGTGTACCTAGATTTTAGATCCCTGTACCCCTCAATCATCATCACCCACAACGTCTCGCCGGATACGCTCAACAGAGAAGGATGCAAGGAATATGACGTTGCCCCACAGGTCGGCCACCGCTTCTGCAAGGACTTCCCAGGATTTATCCCGAGCCTGCTTGGAGACCTCCTAGAGGAGAGGCAGAAGATAAAGAAGAAGATGAAGGCCACGATTGACCCGATCGAGAGGAAGCTCCTCGATTACAGGCAGAGGGCCATCAAGATCCTGGCAAACAGCTACTACGGTTACTACGGCTATGCAAGGGCGCGCTGGTACTGCAAGGAGTGTGCAGAGAGCGTAACGGCCTGGGGAAGGGAGTACATAACGATGACCATCAAGGAGATAGAGGAAAAGTACGGCTTTAAGGTAATCTACAGCGACACCGACGGATTTTTTGCCACAATACCTGGAGCCGATGCTGAAACCGTCAAAAAGAAGGCTATGGAGTTCCTCAAGTATATCAACGCCAAACTTCCGGGCGCGCTTGAGCTCGAGTACGAGGGCTTCTACAAACGCGGCTTCTTCGTCACGAAGAAGAAGTATGCGGTGATAGACGAGGAAGGCAAGATAACAACGCGCGGACTTGAGATTGTGAGGCGTGACTGGAGCGAGATAGCGAAAGAGACGCAGGCGAGGGTTCTTGAAGCTTTGCTAAAGGACGGTGACGTCGAGAAGGCCGTGAGGATAGTCAAAGAAGTTACCGAAAAGCTGAGCAAGTACGAGGTTCCGCCGGAGAAGCTGGTGATCCACGAGCAGATAACGAGGGATTTAAAGGACTACAAGGCAACCGGTCCCCACGTTGCCGTTGCCAAGAGGTTGGCCGCGAGAGGAGTCAAAATACGCCCTGGAACGGTGATAAGCTACATCGTGCTCAAGGGCTCTGGGAGGATAGGCGACAGGGCGATACCGTTCGACGAGTTCGACCCGACGAAGCACAAGTACGACGCCGAGTACTACATTGAGAACCAGGTTCTCCCAGCCGTTGAGAGAATTCTGAGAGCCTTCGGTTACCGCAAGGAAGACCTGCGCTACCAGAAGACGAGACAGGTTGGTTTGAGTGCTTGGCTGAAGCCGAAGGGAACTTGAGTCGACCTGCAGCCAAGCTTAATTAGCTGAGCTTGGACTCCTGTTGATAGATCCAGTAATGACCTCAGAACTCCATCTGGATTTGTTCAGAACGCTCGGTTGCCGCCGGGCGTTTTTTATTGGTGAGAATCCAAGCTAGCTTGGCGAGATTTTCAGGAGCTAAGGAAGCTAAAATGGAGAAAAAAATCACTGGATATACCACCGTTGATATATCCCAATGGCATCGTAAAGAACATTTTGAGGCATTTCAGTCAGTTGCTCAATGTACCTATAACCAGACCGTTCAGCTGGATATTACGGCCTTTTTAAAGACCGTAAAGAAAAATAAGCACAAGTTTTATCCGGCCTTTATTCACATTCTTGCCCGCCTGATGAATGCTCATCCGGAATTTCGTATGGCAATGAAAGACGGTGAGCTGGTGATATGGGATAGTGTTCACCCTTGTTACACCGTTTTCCATGAGCAAACTGAAACGTTTTCATCGCTCTGGAGTGAATACCACGACGATTTCCGGCAGTTTCTACACATATATTCGCAAGATGTGGCGTGTTACGGTGAAAACCTGGCCTATTTCCCTAAAGGGTTTATTGAGAATATGTTTTTCGTCTCAGCCAATCCCTGGGTGAGTTTCACCAGTTTTGATTTAAACGTGGCCAATATGGACAACTTCTTCGCCCCCGTTTTCACCATGGGCAAATATTATACGCAAGGCGACAAGGTGCTGATGCCGCTGGCGATTCAGGTTCATCATGCCGTTTGTGATGGCTTCCATGTCGGCAGAATGCTTAATGAATTACAACAGTACTGCGATGAGTGGCAGGGCGGGGCGTAATTTTTTTAAGGCAGTTATTGGTGCCCTTAAACGCCTGGGGTAATGACTCTCTAGCTTGAGGCATCAAATAAAACGAAAGGCTCAGTCGAAAGACTGGGCCTTTCGTTTTATCTGTTGTTTGTCGGTGAACGCTCTCCTGAGTAGGACAAATCCGCCGCTCTAGATTACCGTGCAGTCGATGATAAGCTGTCAAACATGAGAATTGTGCCTAATGAGTGAGCTAACTCACATTAATTGCGTTGCGCTCACTGCCCGCTTTCCAGTCGGGAAACCTGTCGTGCCAGCTGCATTAATGAATCGGCCAACGCGCGGGGAGAGGCGGTTTGCGTATTGGGCGCCAGGGTGGTTTTTCTTTTCACCAGTGAGACGGGCAACAGCTGATTGCCCTTCACCGCCTGGCCCTGAGAGAGTTGCAGCAAGCGGTCCACGCTGGTTTGCCCCAGCAGGCGAAAATCCTGTTTGATGGTGGTTAACGGCGGGATATAACATGAGCTGTCTTCGGTATCGTCGTATCCCACTACCGAGATATCCGCACCAACGCGCAGCCCGGACTCGGTAATGGCGCGCATTGCGCCCAGCGCCATCTGATCGTTGGCAACCAGCATCGCAGTGGGAACGATGCCCTCATTCAGCATTTGCATGGTTTGTTGAAAACCGGACATGGCACTCCAGTCGCCTTCCCGTTCCGCTATCGGCTGAATTTGATTGCGAGTGAGATATTTATGCCAGCCAGCCAGACGCAGACGCGCCGAGACAGAACTTAATGGGCCCGCTAACAGCGCGATTTGCTGGTGACCCAATGCGACCAGATGCTCCACGCCCAGTCGCGTACCGTCTTCATGGGAGAAAATAATACTGTTGATGGGTGTCTGGTCAGAGACATCAAGAAATAACGCCGGAACATTAGTGCAGGCAGCTTCCACAGCAATGGCATCCTGGTCATCCAGCGGATAGTTAATGATCAGCCCACTGACGCGTTGCGCGAGAAGATTGTGCACCGCCGCTTTACAGGCTTCGACGCCGCTTCGTTCTACCATCGACACCACCACGCTGGCACCCAGTTGATCGGCGCGAGATTTAATCGCCGCGACAATTTGCGACGGCGCGTGCAGGGCCAGACTGGAGGTGGCAACGCCAATCAGCAACGACTGTTTGCCCGCCAGTTGTTGTGCCACGCGGTTGGGAATGTAATTCAGCTCCGCCATCGCCGCTTCCACTTTTTCCCGCGTTTTCGCAGAAACGTGGCTGGCCTGGTTCACCACGCGGGAAACGGTCTGATAAGAGACACCGGCATACTCTGCGACATCGTATAACGTTACTGGTTTCATTCACCACCCTGAATTGACTCTCTTCCGGGCGCTATCATGCCATACCGCGAAAGGTTTTGCACCATTCGATGGTGTCGGAATTTCGGGCAGCGTTGGGTCCTGGCCACGGGTGCGCATGATCTAGAGCTGCCTCGCGCGTTTCGGTGATGACGGTGAAAACCTCTGACACATGCAGCTCCCGGAGACGGTCACAGCTTGTCTGTAAGCGGATGCCGGGAGCAGACAAGCCCGTCAGGGCGCGTCAGCGGGTGTTGGCGGGTGTCGGGGCGCAGCCATGACCCAGTCACGTAGCGATAGCGGAGTGTATACTGGCTTAACTATGCGGCATCAGAGCAGATTGTACTGAGAGTGCACCATATGCGGTGTGAAATACCGCACAGATGCGTAAGGAGAAAATACCGCATCAGGCGCTCTTCCGCTTCCTCGCTCACTGACTCGCTGCGCTCGGTCGTTCGGCTGCGGCGAGCGGTATCAGCTCACTCAAAGGCGGTAATACGGTTATCCACAGAATCAGGGGATAACGCAGGAAAGAACATGTGAGCAAAAGGCCAGCAAAAGGCCAGGAACCGTAAAAAGGCCGCGTTGCTGGCGTTTTTCCATAGGCTCCGCCCCCCTGACGAGCATCACAAAAATCGACGCTCAAGTCAGAGGTGGCGAAACCCGACAGGACTATAAAGATACCAGGCGTTTCCCCCTGGAAGCTCCCTCGTGCGCTCTCCTGTTCCGACCCTGCCGCTTACCGGATACCTGTCCGCCTTTCTCCCTTCGGGAAGCGTGGCGCTTTCTCATAGCTCACGCTGTAGGTATCTCAGTTCGGTGTAGGTCGTTCGCTCCAAGCTGGGCTGTGTGCACGAACCCCCCGTTCAGCCCGACCGCTGCGCCTTATCCGGTAACTATCGTCTTGAGTCCAACCCGGTAAGACACGACTTATCGCCACTGGCAGCAGCCACTGGTAACAGGATTAGCAGAGCGAGGTATGTAGGCGGTGCTACAGAGTTCTTGAAGTGGTGGCCTAACTACGGCTACACTAGAAGGACAGTATTTGGTATCTGCGCTCTGCTGAAGCCAGTTACCTTCGGAAAAAGAGTTGGTAGCTCTTGATCCGGCAAACAAACCACCGCTGGTAGCGGTGGTTTTTTTGTTTGCAAGCAGCAGATTACGCGCAGAAAAAAAGGATCTCAAGAAGATCCTTTGATCTTTTCTACGGGGTCTGACGCTCAGTGGAACGAAAACTCACGTTAAGGGATTTTGGTCATGAGATTATCAAAAAGGATCTTCACCTAGATCCTTTTAAATTAAAAATGAAGTTTTAAATCAATCTAAAGTATATATGAGTAAACTTGGTCTGACAGTTACCAATGCTTAATCAGTGAGGCACCTATCTCAGCGATCTGTCTATTTCGTTCATCCATAGTTGCCTGACTCCCCGTCGTGTAGATAACTACGATACGGGAGGGCTTACCATCTGGCCCCAGTGCTGCAATGATACCGCGgGACCCACGCTCACCGGCTCCAGATTTATCAGCAATAAACCAGCCAGCCGGAAGGGCCGAGCGCAGAAGTGGTCCTGCAACTTTATCCGCCTCCATCCAGTCTATTAATTGTTGCCGGGAAGCTAGAGTAAGTAGTTCGCCAGTTAATAGTTTGCGCAACGTTGTTGCCATTGCTACAGGCATCGTGGTGTCACGCTCGTCGTTTGGTATGGCTTCATTCAGCTCCGGTTCCCAACGATCAAGGCGAGTTACATGATCCCCCATGTTGTGCAAAAAAGCGGTTAGCTCCTTCGGTCCTCCGATCGTTGTCAGAAGTAAGTTGGCCGCAGTGTTATCACTCATGGTTATGGCAGCACTGCATAATTCTCTTACTGTCATGCCATCCGTAAGATGCTTTTCTGTGACTGGTGAGTACTCAACCAAGTCATTCTGAGAATAGTGTATGCGGCGACCGAGTTGCTCTTGCCCGGCGTCAATACGGGATAATACCGCGCCACATAGCAGAACTTTAAAAGTGCTCATCATTGGAAAACGTTCTTCGGGGCGAAAACTCTCAAGGATCTTACCGCTGTTGAGATCCAGTTCGATGTAACCCACTCGTGCACCCAACTGATCTTCAGCATCTTTTACTTTCACCAGCGTTTCTGGGTGAGCAAAAACAGGAAGGCAAAATGCCGCAAAAAAGGGAATAAGGGCGACACGGAAATGTTGAATACTCATACTCTTCCTTTTTCAATATTATTGAAGCATTTATCAGGGTTATTGTCTCATGAGCGGATACATATTTGAATGTATTTAGAAAAATAAACAAATAGGGGTTCCGCGCACATTTCCCCGAAAAGTGCCACCTGACGTCTAAGAAACCATTATTATCATGACATTAACCTATAAAAATAGGCGTATCACGAGGCCCTTTCGTCTTCACCTCGAGAAATCATAAAAAATTTATTTGCTTTGTGAGCGGATAACAATTATAATAGATTCAATTGTGAGCGGATAACAATTTCACACAGAATTCATTAAAGAGGAGAAATTAACTATGAGAGGATCTCACCATCACCATCACCATACGGATCCAAGCGGCCTGGTGCCGCGCGGCAGC
